# Peripheral Blood Biomarkers Predict Outcomes in Advanced Cancers Treated With Anti‐PD‐1 Therapy

**DOI:** 10.1002/iid3.70402

**Published:** 2026-05-06

**Authors:** Chunxia Feng, Ping Li, Qianhui Gu, Minbin Chen, Jinhua Gu

**Affiliations:** ^1^ Department of Radiotherapy and Oncology Affiliated Kunshan Hospital of Jiangsu University Kunshan Jiangsu China; ^2^ Department of Clinical Laboratory Affiliated Kunshan Hospital of Jiangsu University Kunshan Jiangsu China

**Keywords:** advanced cancer, PD‐1 inhibitors, peripheral blood markers, prognostic biomarkers, treatment response prediction

## Abstract

**Objective:**

PD‐1 inhibitors are increasingly used in advanced cancers, but reliable biomarkers for predicting treatment response remain limited.

**Methods:**

In this retrospective cohort study, we analyzed 335 patients with stage IV cancers treated with anti‐PD‐1 therapy (The First People's Hospital of Kunshan, 2019–2025). A predictive model integrating peripheral blood markers and clinical factors was developed using logistic and Cox regression.

**Results:**

Histologic subtype, dNLR ≤ 3.18, AMC ≤ 0.48, and IL‐6 ≤ 12.35 were independent prognostic factors for OS and PFS (multivariate HRs reported). Patients with ≥ 3 favorable factors had significantly better outcomes. The model showed high accuracy for 1‐year survival prediction (AUC = 0.83) but limited performance beyond 3 years.

**Conclusion:**

This study proposes a clinically feasible biomarker‐based tool to stratify PD‐1 inhibitor responders, though further validation in prospective cohorts is needed.

## Graphical abstract

This study identifies dNLR≤ 3.18, AMC ≤ 0.48, IL‐6 ≤ 12.35, and squamous histology as independent predictors of improved survival in advanced cancers treated with anti‐PD‐1 therapy. A novel biomarker‐based model demonstrates high accuracy for 1‐year survival stratification, aiding clinical decision‐making.

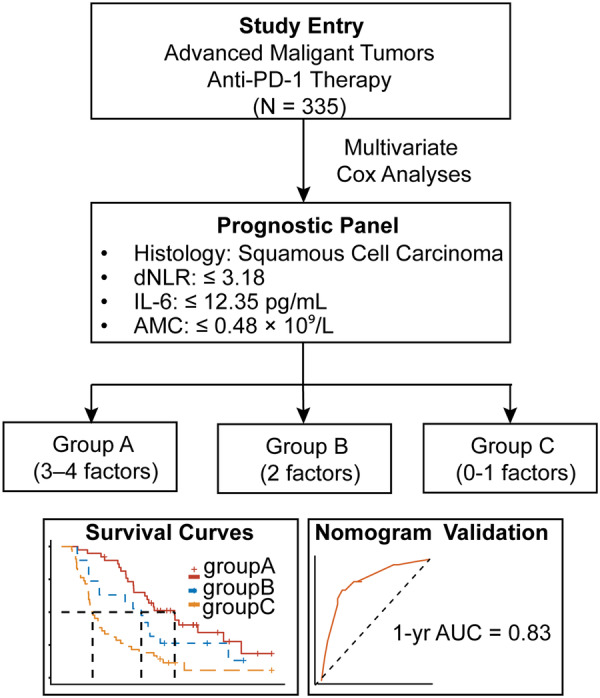

## 1 Introduction

The management of advanced malignant tumors poses a substantial global public health challenge because of their high mortality and limited curative options [1]. In China, the incidence and mortality of advanced cancers such as lung, liver, and pancreatic cancers have increased considerably, posing a severe threat to public health [2]. Advancements in medical technology have introduced immunotherapies, such as programmed cell death‐1 (PD‐1) inhibitors, to patients with advanced cancers by leveraging their immune response against tumors [3, 4]. Integrating PD‐1 inhibitors into standard treatment protocols has emerged as a vital component for the treatment of refractory tumors, highlighting their growing importance in clinical practice [5].

PD‐1 inhibitors enhance the ability of T cells to destroy tumor cells by blocking the PD‐1/Programmed death‐ligand 1 (PD‐L1) signaling pathway, thereby counteracting tumor‐induced immune suppression [4, 5]. Researchers have demonstrated the efficacy of PD‐1 inhibitors for the treatment of advanced malignancies [5, 6]. Clinical trials such as Keynote‐001 [7], Keynote‐042 [8], and CheckMate‐227 [9] have confirmed their efficacy in treating advanced malignancies. However, responses to PD‐1 inhibitors vary considerably, with some patients demonstrating suboptimal responses or developing hyperprogressive disease [5]. Therefore, predicting treatment response to PD‐1 inhibitor therapy and identifying patients most likely to benefit from treatment is critical. Biomarkers in tumor tissues, such as PD‐L1 expression, tumor mutational burden (TMB), and microsatellite instability (MSI), have been extensively studied. Nonetheless, tumor heterogeneity affects their predictive utility, which limits their reliability [5, 10]. Additionally, blood‐based biomarkers, such as T‐cell subsets [11], soluble PD‐L1 [12], and circulating tumor DNA [13] show potential as predictive tools; however, their clinical utility remains expensive and warrants further validation to confirm their reliability and predictive value [14].

The cost‐effectiveness and accessibility of peripheral blood biomarkers have prompted extensive research on their prognostic value in cancer immunotherapy [15, 16, 17]. Recognizing the limitations of individual biomarkers in predicting the efficacy of PD‐1 inhibitors, researchers are focusing on combining peripheral blood parameters into composite indices that can predict immunotherapy response [16]. Furthermore, they have consolidated peripheral blood biomarkers as predictive tools for determining the therapeutic effect of PD‐1 inhibitors [18, 19]. For instance, an elevated baseline‐derived neutrophil‐to‐lymphocyte ratio (dNLR) (> 3) and high levels of lactate dehydrogenase (LDH) (above the upper limit of normal) have been associated with unfavorable outcomes in immune checkpoint inhibitor therapy [19]. Creating a predictive model that incorporates these biomarkers can assist clinicians in daily decision‐making regarding immunotherapy [16, 19]. We aimed to predict the treatment response to PD‐1 inhibitors in patients with advanced malignant tumors by analyzing a panel of 11 peripheral blood markers, along with conventional clinical and pathological characteristics.

This study included 335 patients diagnosed with stage IV malignant tumors. Logistic regression analysis was conducted to explore factors influencing treatment responses. Univariate and multivariate Cox regression analyses were used to assess prognostic markers for overall survival (OS) and progression‐free survival (PFS). Furthermore, a predictive model was developed and validated to categorize patients undergoing PD‐1 inhibitor therapy effectively.

## 2 Methods

### 2.1 Patients

This study included patients with advanced malignant tumors who were treated with anti‐PD‐1 monotherapy or anti‐PD‐1‐based combination therapy at The First People's Hospital of Kunshan between January 2019 and March 2025. The cohort included 335 patients aged ≥ 18 years. All patients had a confirmed pathological diagnosis of stage IV advanced or recurrent malignant tumors. Before treatment (within 0–7 days), routine bloodwork and at least two serum cytokine levels were obtained. Baseline clinical features such as age, sex, Eastern Cooperative Oncology Group Performance Status (ECOG PS), histologic subtype, sites of metastasis, prior treatment history, and treatment methods were recorded. Additional inclusion criteria were as follows: (1) administration of at least two cycles of PD‐1 inhibitor therapy, (2) at least one measurable lesion according to the Response Evaluation Criteria in Solid Tumors (RECIST) version 1.1, (3) baseline computed tomography (CT) or magnetic resonance imaging (MRI) assessment within 4 weeks before initiating PD‐1 inhibitors, (4) ECOG PS ranging from 0 to 2, without substantial comorbidities, and (5) absence of uncontrolled infections. The final follow‐up date was March 31, 2025.

### 2.2 Data Collection

Patients who met the inclusion criteria received PD‐1 inhibitors via intravenous infusions every 21 days, per medical instructions, until death or onset of intolerable toxicities. During disease progression, the treatment can be extended to provide clinical benefits. Peripheral blood data were collected, including the following parameters: absolute neutrophil count (ANC) (×10^9^/L), absolute lymphocyte count (ALC) (×10^9^/L), absolute monocyte count (AMC) (×10^9^/L), serum albumin (g/L), LDH (U/L), interleukin‐6 (IL‐6) levels (pg/mL), and IL‐10 levels (pg/mL). The following composite indices were calculated: (1) neutrophil‐to‐lymphocyte ratio (NLR): ANC ÷ ALC, (2) dNLR: ANC/(white cell count ‐ ANC), (3) platelet‐to‐lymphocyte ratio (PLR): platelet count ÷ ALC, and (4) prognostic nutritional index (PNI): serum albumin (g/L) + 5 × ALC (×10^9^/L). Patients with incomplete baseline blood marker data or lost to follow‐up were excluded from the analysis. This study was conducted in compliance with the principles of Good Clinical Practice and the Declaration of Helsinki (revised in 2013). This study was approved by the Institutional Review Board of the First People's Hospital of Kunshan (ethical approval number: 2023‐06‐012), and the need for individual informed consent was waived due to the retrospective nature of the analysis.

### 2.3 Response Assessments

Patient responses were assessed using the RECIST 1.1 guidelines every 6–8 weeks after initiating PD‐1 inhibitor therapy, utilizing CT or MRI scans. The best overall response during treatment was classified as complete response (CR), partial response (PR), stable disease (SD), or progressive disease (PD). The patients were divided into two groups: (i) the clinically beneficial disease (CD) group, including patients with CR, PR, or SD, and (ii) the PD group. The overall response rate (ORR) was calculated as follows: (CR + PR)/total number of patients × 100%. The disease control rate (DCR) was calculated as follows: (CR + PR + SD)/total number of patients × 100%.

OS was defined as the time from the initial administration of PD‐1 inhibitors to either the date of death or final clinical assessment. PFS was calculated from the date of initial administration of PD‐1 inhibitors to either disease progression or the last clinical assessment. PFS was considered equivalent to OS in patients who died before disease progression.

### 2.4 Statistical Analysis

This study summarized patient characteristics using descriptive statistics. Categorical variables were presented as numbers and percentages, whereas continuous variables were presented as medians and interquartile ranges. The *χ* ^2^ test was used to compare categorical variables, whereas the Kruskal–Wallis *H* and Student's *t*‐tests were used to compare continuous variables. Kaplan–Meier analysis was conducted to evaluate PFS and OS based on specified cutoff values, and the log‐rank test was used to determine differences between groups. Furthermore, univariate Cox models were applied to identify factors associated with OS and PFS, with significant variables (*p* < 0.1) included in the multivariate Cox analysis. Outcomes are expressed as hazard ratios (HR) and 95% confidence intervals (CI); a *p*‐value < 0.05 indicated statistical significance. Data analysis and visualization were performed using SPSS (Version 25.0. Armonk, NY, USA) and R software (version 4.3.3; R Foundation for Statistical Computing, Vienna, Austria).

## 3 Results

### 3.1 Patient Characteristics

We investigated the treatment outcomes of 352 patients with advanced malignant tumors who received anti‐PD‐1 monotherapy or anti‐PD‐1‐based combination therapy at The First People's Hospital of Kunshan between January 2019 and March 2025. Of all the patients, 335 provided routine blood test results and clinical information before treatment. The cohort consisted of patients with various tumor types, including lung cancer (24.2%), gastric cancer (12.2%), liver cancer (11.9%), head and neck malignancies (10.4%), colorectal cancer (9%), esophageal cancer (8.4%), cholangiocarcinoma (4.2%), pancreatic cancer (4.2%), cervical cancer (3.9%), urinary tract tumors (3.9%), breast cancer (1.2%), ovarian cancer (0.9%), and other cancers (5.7%). Among the 335 patients, 76.4% had an ECOG PS of 0 to 1, 56.1% were diagnosed with adenocarcinoma, and 30.1% were diagnosed with squamous cell carcinoma. Metastasis to more than two organs was observed in 17.3% of the patients, with lymph nodes being the most common metastasis site (49.6%) (Table 1).

**Table 1:** Patient characteristics and the association between Radiological Response and clinical categorical variables.

Characteristics	Level	Overall	CD	PD	*p*	SMD
*N*		335	247	88		
Age (%)	≤ 73	282 (84.2)	211 (85.4)	71 (80.7)	0.381	0.127
	> 73	53 (15.8)	36 (14.6)	17 (19.3)		
Gender (%)	woman	122 (36.4)	85 (34.4)	37 (42.0)	0.251	0.158
	man	213 (63.6)	162 (65.6)	51 (58.0)		
ECOG performance status (%)	0–1	256 (76.4)	190 (76.9)	66 (75.0)	0.827	0.045
	≥ 2	79 (23.6)	57 (23.1)	22 (25.0)		
Primary cancer sites (%)	Biliary duct	14 (4.2)	11 (4.5)	3 (3.4)	0.292	0.461
	Breast	4 (1.2)	3 (1.2)	1 (1.1)		
	Cervix	13 (3.9)	8 (3.2)	5 (5.7)		
	Colorectal	30 (9.0)	23 (9.3)	7 (8.0)		
	Esophagus	28 (8.4)	22 (8.9)	6 (6.8)		
	Gastric	41 (12.2)	27 (10.9)	14 (15.9)		
	Head and neck	35 (10.4)	30 (12.1)	5 (5.7)		
	Liver	40 (11.9)	24 (9.7)	16 (18.2)		
	Lung	81 (24.2)	65 (26.3)	16 (18.2)		
	Others	19 (5.7)	13 (5.3)	6 (6.8)		
	Ovary	3 (0.9)	1 (0.4)	2 (2.3)		
	Pancreas	14 (4.2)	10 (4.0)	4 (4.5)		
	Urinary	13 (3.9)	10 (4.0)	3 (3.4)		
Histologic subtype (%)	Adenocarcinoma	188 (56.1)	132 (53.4)	56 (63.6)	0.069	0.3
	Others	46 (13.7)	32 (13.0)	14 (15.9)		
	Squamous carcinoma	101 (30.1)	83 (33.6)	18 (20.5)		
Treatment Lines (%)	1–2	249 (74.3)	180 (72.9)	69 (78.4)	0.38	0.129
	≥ 3	86 (25.7)	67 (27.1)	19 (21.6)		
Number of metastatic sites (%)	1–2	277 (82.7)	205 (83.0)	72 (81.8)	0.931	0.031
	≥ 3	58 (17.3)	42 (17.0)	16 (18.2)		
Bone (%)	Non‐metastatic	247 (73.7)	183 (74.1)	64 (72.7)	0.914	0.031
	Metastasis	88 (26.3)	64 (25.9)	24 (27.3)		
Lung (%)	Non‐metastatic	246 (73.4)	182 (73.7)	64 (72.7)	0.973	0.022
	Metastasis	89 (26.6)	65 (26.3)	24 (27.3)		
Liver (%)	Non‐metastatic	238 (71.0)	173 (70.0)	65 (73.9)	0.588	0.085
	Metastasis	97 (29.0)	74 (30.0)	23 (26.1)		
Lymph node (%)	Non‐metastatic	169 (50.4)	123 (49.8)	46 (52.3)	0.784	0.05
	Metastasis	166 (49.6)	124 (50.2)	42 (47.7)		
Anti‐PD‐1 cycles (median [IQR])		6.00 [4.00, 13.00]	8.00 [4.00, 15.50]	4.00 [2.75, 6.00]	< 0.001	0.771
Treatment method (%)	Anti‐PD‐1 group	72 (21.5)	53 (21.5)	19 (21.6)	0.981	0.052
	Anti‐PD‐1 plus chemotherapy group	150 (44.8)	110 (44.5)	40 (45.5)		
	Anti‐PD‐1 plus targeted therapy group	96 (28.7)	72 (29.1)	24 (27.3)		
	Triple group	17 (5.1)	12 (4.9)	5 (5.7)		
Response (%)	CR	11 (3.3)	11 (4.5)	0 (0.0)	< 0.001	6.551
	PD	88 (26.3)	0 (0.0)	88 (100.0)		
	PR	55 (16.4)	55 (22.3)	0 (0.0)		
	SD	181 (54.0)	181 (73.3)	0 (0.0)		

Abbreviations: CD, Clinically Beneficial Disease Group; ECOG, Eastern Cooperative Oncology Group; IQR, interquartile range; PD, Progressive Disease Group.

Regarding treatment, 21.5% of patients received PD‐1 inhibitor monotherapy, whereas 78.5% received combination therapy, which included chemotherapy, targeted therapy, or both. Among the 249 patients (74.3%) who received PD‐1 inhibitors as first‐ and second‐line treatment, 32.2% received first‐line therapy (Table 1). The median follow‐up time was 28.4 months (95% CI: 26.6–32.9). The median OS was 16.2 months (95% CI: 14.1–19.1), and the median PFS was 10.4 months (95% CI: 8.6–12.7). For the entire cohort, the DCR was 73.73% and ORR was 19.70%. For patients who received PD‐1 inhibitor monotherapy, the ORR was 26.37% and DCR was 73.61%. In contrast, for patients who received combination therapy, the ORR was 17.87% and DCR was 73.76% (Table S1).

### 3.2 Radiological Response and Baseline Peripheral Blood Immune Features

**Figure 1:**
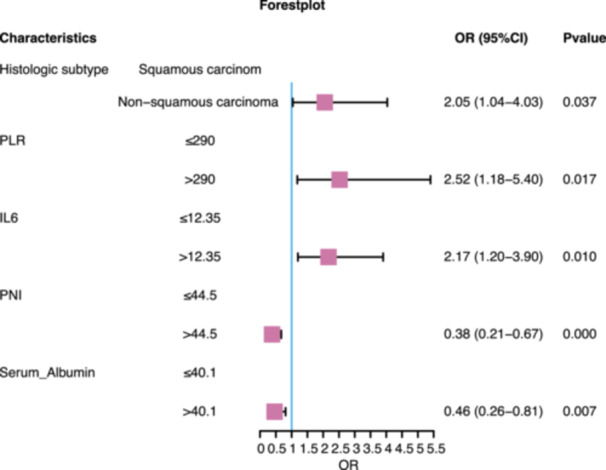
Association between baseline patient characteristics and radiological response (*n* = 335). The forest plot illustrates the influence of positive baseline biomarkers on clinical response. The odds ratio (OR) and corresponding 95% confidence intervals (CIs) were derived from univariate logistic regression analysis. The vertical line at OR = 1 distinguishes the OR values predicting clinically beneficial disease (CD) (left side) from those predicting progressive disease (PD) (right side).

The CD group had better OS and PFS than the PD group (Figure S1). We examined the predictive value of baseline peripheral blood biomarkers for determining the clinical response to PD‐1 inhibitors in patients with advanced malignant tumors. Patients in the PD group underwent fewer cycles of immunotherapy than patients in the CD group (PD vs. CD: 4.00 [2.75, 6.00] cycles vs. 8.00 [4.00, 15.50] cycles; Table 1). Furthermore, the nonsquamous histological subtype was associated with a higher probability of disease progression (Figure 1). Additionally, key biomarkers, including elevated PLR (> 290), increased IL6 levels (> 12.35), reduced PNI (≤ 44.5), and decreased serum albumin levels (≤ 40.1), were associated with disease progression (Figure 1). Histological subtype emerged as a crucial independent determinant of clinical outcomes, underscoring its critical role in disease progression (Supplementary Table 2). These findings highlight the importance of utilizing peripheral blood markers to assess clinical response to anti‐PD‐1 therapy.

### 3.3 Univariable and Multivariable Analysis for Survival Outcome

**Figure 2:**
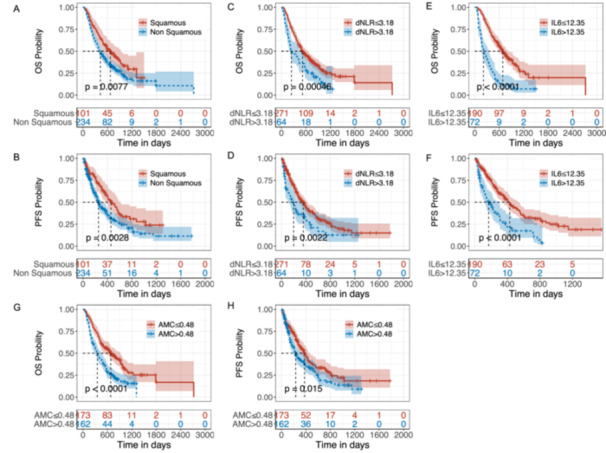
Kaplan–Meier survival analysis of overall survival (OS) and progression‐free survival (PFS) by pathological type and peripheral blood biomarkers. (A and B) Survival curves comparing squamous cell carcinoma with nonsquamous cell carcinoma. (C and D) Survival curves based on derived neutrophil‐to‐lymphocyte ratio (dNLR) cut‐off values. (E and F) Survival curves based on interleukin‐6 (IL‐6) cut‐off values. (G and H) Survival curves based on absolute monocyte count (AMC) cut‐off values. P‐values are determined using the log‐rank test.

We analyzed 11 peripheral blood parameters measured before treatment to explore their effects on OS and PFS after PD‐1 inhibitor therapy. Univariate Cox proportional hazards regression analysis suggested that ECOG PS ≥ 2, nonsquamous carcinomas, treatment beyond the third line, liver metastasis, NLR > 4.48, dNLR > 3.18, IL‐6 > 12.35, IL‐10 > 4.96, AMC > 0.48, serum albumin ≤ 40.1, LDH > 250, and PNI ≤ 44.5 were associated with shorter OS and PFS (Figure 2, Figure S2, S3, Tables 2 and 3). Additionally, poor OS was associated with pretreatment levels of age > 73 years, PLR > 290, ANC > 5.34, and ALC ≤ 1.46 (Table 2). Moreover, women with three or more metastatic sites had significantly worse PFS (*p* < 0.05) (Table 3).

**Table 2:** Univariable and multivariable analysis of OS.

Characteristics	Level	Univariate HR	95% CI	*p* value	Multivariate HR	95% CI	*p* value
Age	> 73 y (vs. ≤ 73 y)	1.42	1.01–1.98	0.041	0.95	0.61–1.46	0.809
Gender	Woman (vs. man)	0.98	0.75–1.28	0.861			
ECOG performance status	≥ 2 (vs. 0–1)	1.46	1.1–1.95	0.009	1.11	0.75–1.64	0.59
Histologic subtype	Non‐squamous carcinoma (vs. Squamous carcinoma)	1.49	1.11–2	0.008	1.6	1.09–2.35	0.016
Treatment Lines	≥ 3 (vs. 1–2)	1.36	1.02–1.8	0.035	1.27	0.87–1.86	0.214
Number of metastatic sites	≥ 3 (vs. 1–2)	1.23	0.89–1.7	0.208			
Bone	Metastasis (vs. non‐metastatic)	1.08	0.81–1.43	0.613			
Lung	Metastasis (vs. non‐metastatic)	1.27	0.96–1.69	0.092	1.5	1.07–2.11	0.018
Liver	Metastasis (vs. non‐metastatic)	1.32	1–1.73	0.049	1.12	0.79–1.59	0.537
Lymph_node	Metastasis (vs. non‐metastatic)	0.84	0.65–1.09	0.197			
NLR	> 4.84 (vs. ≤ 4.84)	1.72	1.29–2.29	0	0.43	0.21–0.87	0.019
dNLR	> 3.18 (vs. ≤ 3.18)	1.72	1.26–2.34	0.001	1.97	1.02–3.8	0.043
PLR	> 290 (vs. ≤ 290)	1.62	1.13–2.32	0.009	1.69	0.99–2.88	0.053
IL6	> 12.35 (vs. ≤ 12.35)	3.05	2.24–4.15	0	2.77	1.95–3.94	0
IL10	> 4.96 (vs. ≤ 4.96)	1.87	1.23–2.84	0.003	1.1	0.69–1.75	0.678
ANC	> 5.34 (vs. ≤ 5.34)	1.61	1.18–2.21	0.003	1.28	0.78–2.1	0.33
ALC	> 1.46 (vs. ≤ 1.46)	0.73	0.55–0.97	0.029	0.65	0.45–0.95	0.028
AMC	> 0.48 (vs. ≤ 0.48)	1.86	1.43–2.41	0	1.71	1.22–2.41	0.002
Serum Albumin	> 40.1 (vs. ≤ 40.1)	0.38	0.29–0.5	0	0.55	0.35–0.86	0.01
LDH	> 250 (vs. ≤ 250)	1.64	1.24–2.16	0	1.21	0.87–1.67	0.263
PNI	> 44.5 (vs. ≤ 44.5)	0.43	0.33–0.55	0	0.96	0.59–1.58	0.882

Abbreviations: ALC, absolute lymphocyte count; AMC, absolute monocyte count; ANC, absolute neutrophil count; dNLR, derived neutrophil‐to‐lymphocyte ratio; ECOG PS, Eastern Cooperative Oncology Group Performance Status; LDH, lactate dehydrogenase; NLR, neutrophil‐to‐lymphocyte ratio; PLR, platelet‐to‐lymphocyte ratio; PNI, prognostic nutritional index.

**Table 3:** Univariable and multivariable analysis of PFS.

Characteristics	Level	Univariate HR	95% CI	*p* value	Multivariate HR	95% CI	*p* value
Age	> 73 y (vs. ≤ 73 y)	0.97	0.66–1.42	0.871			
Gender	Woman (vs. man)	1.38	1.05–1.83	0.021	1.58	1.13–2.22	0.008
ECOG performance status	≥ 2 (vs. 0–1)	1.59	1.15–2.19	0.005	1	0.67–1.51	0.981
Histologic subtype	Non‐squamous carcinoma (vs. Squamous carcinoma)	1.61	1.18–2.2	0.003	1.51	1.01–2.26	0.045
Treatment Lines	≥ 3 (vs. 1–2)	1.45	1.08–1.95	0.015	1.57	1.06–2.33	0.024
Number of metastatic sites	≥ 3 (vs. 1–2)	1.44	1.03–2.01	0.033	1.32	0.86–2.02	0.202
Bone	Metastasis (vs. non‐metastatic)	1.01	0.75–1.38	0.929			
Lung	Metastasis (vs. non‐metastatic)	1.2	0.89–1.62	0.241			
Liver	Metastasis (vs. non‐metastatic)	1.41	1.06–1.89	0.019	1.29	0.89–1.88	0.185
Lymph_node	Metastasis (vs. non‐metastatic)	0.86	0.65–1.13	0.284			
NLR	> 4.84 (vs. ≤ 4.84)	1.73	1.28–2.35	0	0.46	0.22–0.96	0.039
dNLR	> 3.18 (vs. ≤ 3.18)	1.68	1.2–2.35	0.002	2.06	1.01–4.21	0.047
PLR	> 290 (vs. ≤ 290)	1.45	0.98–2.15	0.063	1.69	0.97–2.93	0.064
IL6	> 12.35 (vs. ≤ 12.35)	2.29	1.64–3.2	0	1.86	1.27–2.73	0.002
IL10	> 4.96 (vs. ≤ 4.96)	1.97	1.25–3.11	0.004	1.26	0.76–2.11	0.37
ANC	> 5.34 (vs. ≤ 5.34)	1.29	0.92–1.83	0.145	1.01	0.57–1.79	0.968
ALC	> 1.46 (vs. ≤ 1.46)	0.76	0.56–1.03	0.074	0.7	0.47–1.04	0.078
AMC	> 0.48 (vs. ≤ 0.48)	1.4	1.07–1.85	0.016	1.5	1.04–2.17	0.031
Serum Albumin	> 40.1 (vs. ≤ 40.1)	0.51	0.38–0.67	0	0.67	0.4–1.11	0.119
LDH	> 250 (vs. ≤ 250)	1.44	1.07–1.94	0.015	1.11	0.78–1.59	0.552
PNI	> 44.5 (vs. ≤ 44.5)	0.53	0.4–0.7	0	0.88	0.52–1.51	0.647

Abbreviations: ALC, absolute lymphocyte count; AMC, absolute monocyte count; ANC, absolute neutrophil count; dNLR, derived neutrophil‐to‐lymphocyte ratio; ECOG PS, Eastern Cooperative Oncology Group Performance Status; LDH, lactate dehydrogenase; NLR, neutrophil‐to‐lymphocyte ratio; PLR, platelet‐to‐lymphocyte ratio; PNI, prognostic nutritional index.

Significant covariates (*p* < 0.1) identified in the univariate analysis were evaluated using multivariate Cox regression analysis to identify independent prognostic factors for PD‐1 inhibitor outcomes. Histologic subtype, lung metastasis status, dNLR, IL‐6, ALC, AMC, and serum albumin levels were independent prognostic factors for OS (Table 2). Sex, histological subtype, treatment line, dNLR, IL‐6 level, and AMC were independent prognostic factors for PFS (Table 3). Collectively, dLR, IL‐6, and AMC levels along with histologic subtype serve as independent prognostic factors for both OS and disease‐free survival (DFS).

### 3.4 Multifactor Model for Survival of Patients Receiving PD‑1 Inhibitors

**Figure 3:**
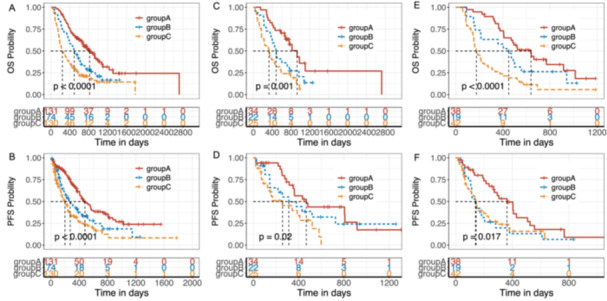
Kaplan–Meier curves illustrating overall survival (OS) and progression‐free survival (PFS) in relation to the number of favorable baseline factors. Favorable baseline factors included squamous cell carcinoma, derived neutrophil‐to‐lymphocyte ratio (dNLR) ≤ 3.18, absolute monocyte count (AMC) ≤ 0.48, and interleukin‐6 (IL‐6) ≤ 12.35. The patients were divided into three groups: Group A (with three or more factors), Group B (with two factors), and Group C (with one or no factors). (A and B) Multifactorial model outcomes for the entire cohort (*N* = 335). (C and D) Subgroup analysis of patients with lung cancer (*N* = 81). (E and F) Subgroup analysis of the patients with gastrointestinal cancer (*n* = 99). *p* values were determined using the log‐rank test.

We further evaluated the prognostic impact of favorable factors, including squamous cell carcinoma, dNLR ≤ 3.18, AMC ≤ 0.48, and IL6 ≤ 12.35, on prognosis. Patients were categorized into three groups based on the number of favorable factors as follows: Group A (*n* = 131, 39.10%) with three to four favorable factors, Group B (*n* = 74, 22.09%) with two favorable factors, and Group C (*n* = 130, 38.81%) with one or no favorable factors. Patients in group A exhibited better OS and DFS than Groups B and C (Figure 3A,B). Multivariate analysis, including clinically relevant covariates, confirmed that the number of favorable factors significantly influenced PFS and OS (Table S3).

Subgroup analysis for essential sample categories, such as lung cancer and malignant tumors of the digestive system (esophagus, stomach, and intestines), demonstrated the efficacy of the model in stratifying patients. Group A demonstrated better OS and PFS than Groups B and C (Figure 3C,D,E,F). Furthermore, stratification efficacy was observed across other subgroups, including patients receiving anti‐PD‐1 monotherapy or combination therapy (Figure S4), patients of different sexes (Figure S5), ECOG PS scores (Figure S6), histological subtypes (Figure S7), metastatic sites (Figure S8), and treatment lines (Figure S9).

The model demonstrated stratification performance according to clinical responses. A higher proportion of patients in group A was observed in the CD group than in the PD group (41.70% vs. 31.81%, Figure S10). Group A demonstrated superior ORR (Figure S11A) and DCR (Figure S11B), compared with Groups B and C. Additionally, patients in group A achieved better clinical outcomes across both the CD and PD groups (Figure S12). Thus, patients with three to four favorable factors (Group A) exhibited significantly improved OS and PFS compared to patients with fewer favorable factors.

### 3.5 Establishing and Validating the Normogram Model

**Figure 4:**
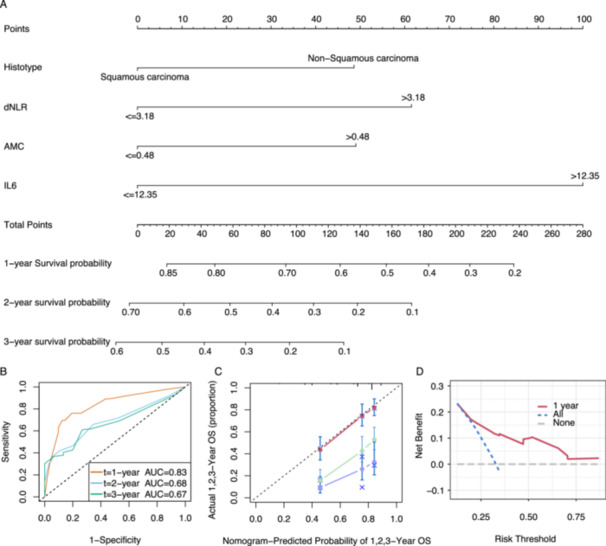
Performance of the nomogram model based on pathological type, derived neutrophil‐to‐lymphocyte ratio (dNLR), absolute monocyte count (AMC), and interleukin‐6 (IL‐6). (A) Nomogram for 1‐, 2‐, and 3‐year survival predictions. (B) Time‐receiver operating characteristic curve at 1‐, 2‐, and 3‐year survival predictions. (C) Calibration plot assessing the predictive accuracy for 1‐, 2‐, and 3‐year survival rates. (D) Curve analysis assessing the clinical utility of the model.

We developed a predictive model incorporating dNLR, IL6, AMC, and histological subtypes. A nomogram was developed to facilitate clinical application (Figure 4A). The model demonstrated excellent predictive ability for 1‐year survival (AUC = 0.83; Figure 4B) and diminished predictive ability for 3‐ and 5‐year survival rates. The calibration plot (Figure 4C) and decision curve analysis (Figure 4D) validated its clinical utility in short‐term survival prediction, potentially enhancing clinical decision making. This nomogram serves as a useful tool for clinicians to evaluate the 1‐year survival probability of patients receiving PD‐1 inhibitor therapy for advanced cancers.

## 4 Discussion

The advantages of tumor tissue biomarkers such as PD‐L1 expression, TMB, and MSI for predicting PD‐1 inhibitor efficacy have been limited by inconsistent testing approaches, high costs, and technical intricacies [14, 20]. In contrast, peripheral blood biomarkers are gaining interest as prognostic tools in cancer immunotherapy because of their accessibility and cost efficiency [11, 15, 16, 18, 21, 22]. We explored the potential of peripheral blood biomarkers to predict responses to PD‐1 inhibitor therapy in advanced cancer to develop a practical and cost‐effective clinical tool for optimizing treatment options.

This study was one of the most extensive single‐center analyses, including 335 patients with advanced malignant tumors. We uniquely identified nonsquamous histology, elevated PLR, increased IL‐6 level, lower PNI, and decreased serum albumin level as predictors of disease progression. By integrating 11 peripheral blood biomarkers with standard clinical factors such as age, sex, and histological subtype, a robust predictive model was developed. Pretreatment levels of dNLR, AMC, and IL‐6, along with histological subtype, serve as independent predictors of PFS and OS. Patients who met the pretreatment criteria of a dNLR ≤ 3.18, AMC ≤ 0.48, IL‐6 ≤ 12.35, and squamous cell carcinoma diagnosis demonstrated significantly longer PFS and OS. These findings lay the foundation for individualized treatment plans for advanced malignancies and support the reliability of this predictive model for estimating 1‐year survival rates.

The role of neutrophils in the tumor microenvironment necessitates careful consideration [16, 23, 24]. Neutrophils are central to tumor progression; they facilitate stromal remodeling, metastasis, angiogenesis, and thrombosis, while suppressing T cell‐mediated antitumor immunity [14, 25]. Our findings elucidate the dNLR as a comprehensive and reliable peripheral blood parameter for prognosis assessment in metastatic disease. dNLR is less influenced by treatment modalities [11, 14]. Elevated dNLR has been correlated with poor survival outcomes in various tumor types, including non‐small cell lung cancer [11, 26], pancreatic cancer [27], bladder cancer [21], and renal cancer [28]. Furthermore, low dNLR levels have been associated with reduced numbers of CD8 +, PD‐1 +, and PD‐1 + CD8 + T cells within the tumor microenvironment, leading to decreased tumor immune infiltration [11]. Patients with a baseline dNLR < 2.6 may benefit from enhanced prognosis when treated with pembrolizumab monotherapy, potentially avoiding the additional toxicity of combined immunotherapy and chemotherapy [11].

Monocytes may influence the effectiveness of PD‐1 inhibitors, affecting T cell function and tumor cell interactions within the microenvironment [29]. Baseline AMC serves as a pan‐cancer biomarker for PD‐1/PD‐L1 inhibitors, with high levels correlating with unfavorable treatment outcomes [30, 31, 32]. Peripheral blood monocytes can infiltrate tissues and differentiate into tumor‐associated macrophages (TAMs), which act as immunosuppressive cells [33] facilitating immune evasion by cancer [34, 35]. High TAM density within the tumor stroma has been associated with a poor prognosis in various cancers [36]. Shao et al. hypothesized that monocyte migration from peripheral blood to tissues create a “hot tumor” microenvironment, enhancing the efficacy of PD‐1/PD‐L1 inhibitors and decreasing circulating AMC levels [30]. Further studies are required to confirm this hypothesis.

Cytokines, small soluble proteins secreted by immune or tumor cells, are central to biological activities including carcinogenesis, suppression of antitumor immunity, and tumor dissemination [37, 38]. Our study, along with prior research [39] has highlighted the association between baseline IL‐6 levels and PD‐1 inhibitor efficacy. Although our findings indicate that elevated IL‐6 levels are associated with reduced survival, the contrasting findings by Yamazaki et al. [39] warrant further investigation. Nonetheless, baseline IL‐6 levels are a potent prognostic biomarker for PD‐1 inhibitor therapy [37, 38, 39].

Finally, we investigated the effect of traditional clinical factors on the efficacy of PD‐1 inhibitors. Patients with nonsquamous cell carcinoma demonstrated inferior treatment outcomes compared to those with squamous cell carcinoma. Clinical trials have reported higher response rates to checkpoint inhibitors in patients with squamous cell carcinoma [40, 41]. Additionally, PD‐L1 expression has been identified as a reliable predictor of response in nonsquamous carcinoma [42].

In this study, a composite model was developed to predict the efficacy of PD‐1 inhibitors using four baseline biomarkers. Patients with advanced malignant tumors who exhibited a dNLR ≤ 3.18, AMC ≤ 0.48, IL‐6 level ≤ 12.35, and squamous cell carcinoma diagnosis demonstrated superior clinical outcomes. Specifically, patients possessing three or more favorable prognostic factors demonstrated a 58% reduction in the risk of disease progression and a 65% reduction in the risk of death compared with patients possessing one or no factors. These findings emphasize the value of a multi‐biomarker approach to enhance the precision and scientific rigor of clinical decision making.

Our observations are supported by previous research. Tanizaki et al. [16] rreported that PD‐L1 expression alone was inadequate for identifying all patients who could benefit from nivolumab therapy. They proposed that a combination of three independent prognostic factors, ANC, ALC, and AEC, could identify patient subgroups that are less likely to respond to nivolumab [16]. Similarly, Peng et al. [22] reported that integrating multiple favorable prognostic factors enhanced the optimization of treatment responses. Our study suggests that stratification based on favorable factors effectively identifies patients with advanced malignancies who are likely to experience positive outcomes after PD‐1 inhibitor therapy. This approach holds promise in advancing personalized treatment strategies in oncology.

Although the present study focuses on the prognostic value of peripheral blood inflammatory markers, emerging evidence suggests that host trace element profiles may offer complementary prognostic information. For instance, a large‐scale study by Jan et al. [43] involving 1475 patients with various cancers demonstrated that higher baseline serum levels of selenium (HR = 0.66) and zinc (HR = 0.55) were independently associated with reduced all‐cause mortality, whereas elevated copper (HR = 1.91) correlated with increased mortality. These elements may interact with inflammatory markers through shared biological pathways, such as the regulation of oxidative stress [43, 44], T‐cell function modulation [45], and angiogenesis [43, 46]. Therefore, integrating trace element status could enhance the multifactorial assessment of patient outcomes.

Nonetheless, this study had some limitations. The relatively small number of patients with specific cancer types and the retrospective, single‐center study design warrant further research to validate our findings in more extensive and diverse patient cohorts. Furthermore, the absence of a control group limits the generalizability of the prognostic value of peripheral blood biomarkers. Additionally, the retrospective design precluded the inclusion of trace element analysis, which could provide additional prognostic insights alongside inflammatory markers. However, the composite model demonstrated strong predictive accuracy for the 1‐year survival rate in patients with advanced cancer. Future research should focus on prospective, randomized, large‐scale clinical trials with robust data to validate our findings.

## 5 Conclusions

In patients with advanced cancers receiving PD‐1 inhibitor therapy, baseline peripheral blood biomarkers such as dNLR, AMC, IL‐6, and tumor histology independently influenced OS and PFS. By integrating these peripheral blood biomarkers with traditional clinical characteristics, a novel prognostic index was developed to identify patients likely to benefit from immunotherapy. The prognostic model exhibited high predictive accuracy for 1‐year survival rates, providing clinicians with a robust tool to optimize treatment strategies and enhance their cost‐effectiveness. Despite the limited sample size, our findings offer a strong scientific rationale for validating the predictive potential of these biomarkers for immunotherapy. In addition, we will integrate a broader range of clinical data into the prognostic value of peripheral blood biomarkers.

## Author Contributions

**Jinhua Gu:** investigation and methodology. **Chunxia Feng** and **Ping Li:** data processing, software, writing draft, and manuscript. **Qianhui Gu** and **Minbin Chen:** visualization, data curation, and supervision.

## Ethics Statement

The Ethics Committee of the First People's Hospital of Kunshan approved this study (approval number: 2023‐06‐012). The need for individual informed consent was waived because of the retrospective nature of the analysis.

## Conflicts of Interest

The authors declare no conflicts of interest.

## Supporting information

Supporting File 1

Supporting File 2

Supporting File 3

Supporting File 4

Supporting File 5

Supporting File 6

Supporting File 7

Supporting File 8

Supporting File 9

Supporting File 10

Supporting File 11

Supporting File 12

Supporting File 13

Supporting File 14

Supporting File 15

## Data Availability

The data that support the findings of this study are available from the corresponding author upon reasonable request.
