# Supplementary figures and images for: Peripheral Blood Biomarkers Predict Outcomes in Advanced Cancers Treated With Anti‐PD‐1 Therapy
Source: Immun Inflamm Dis. 2026 May 6;14(5):e70402. doi: 10.1002/iid3.70402 (PMC13149763; doi:10.1002/iid3.70402)

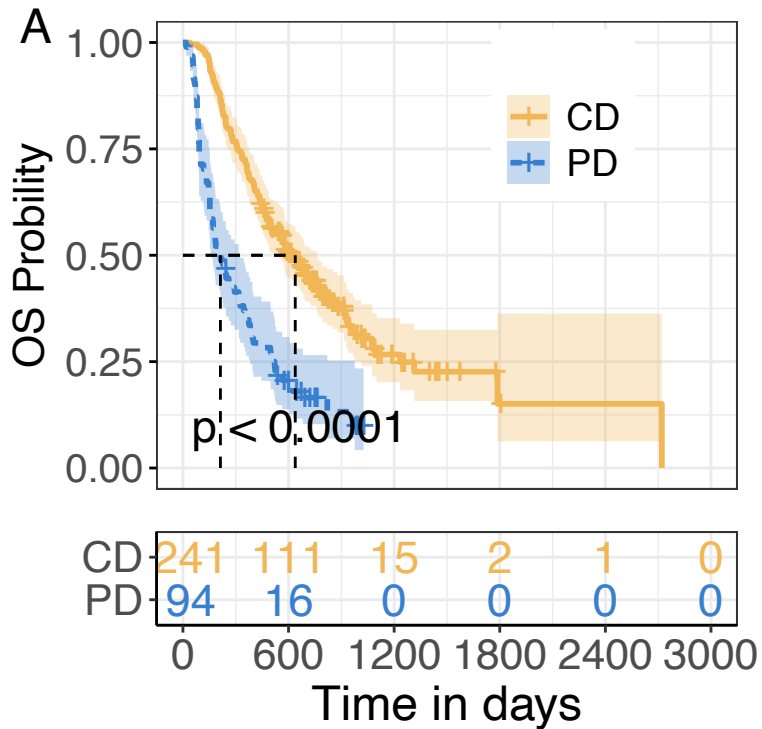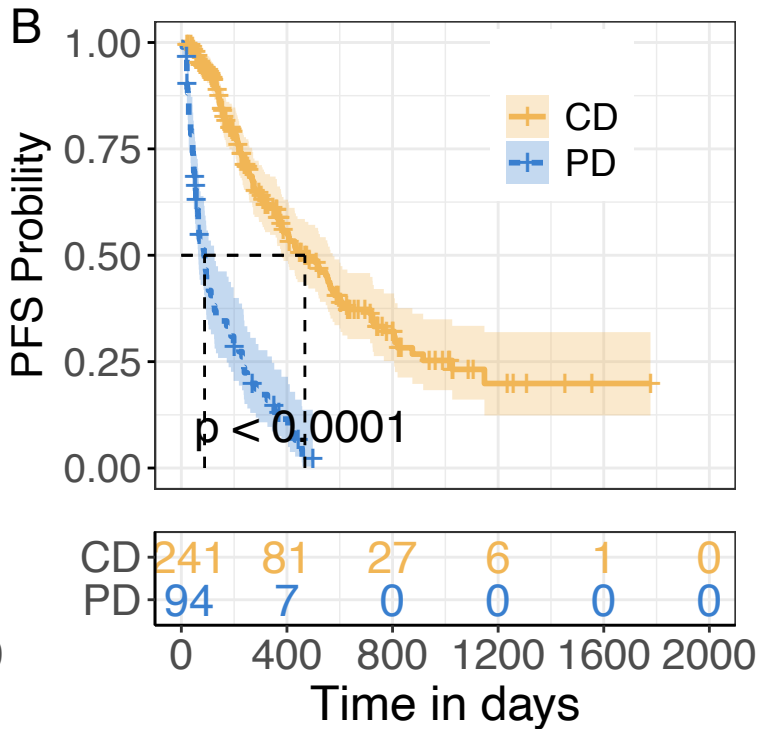

Supplement: Supplementary file 2 — Supporting File 2 [file IID3-14-e70402-s014.pdf]

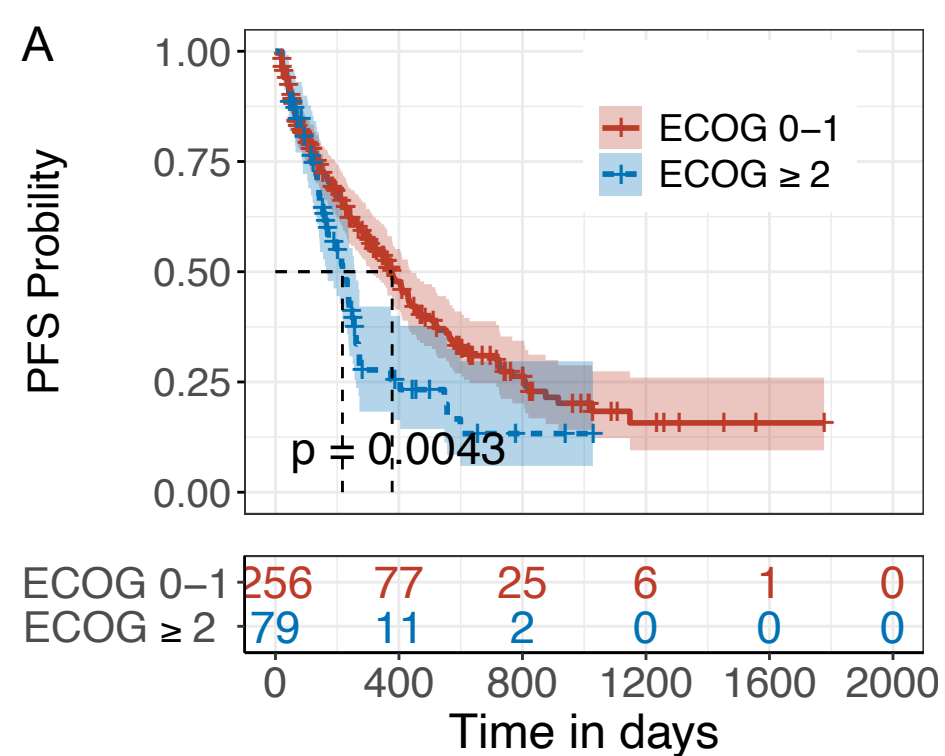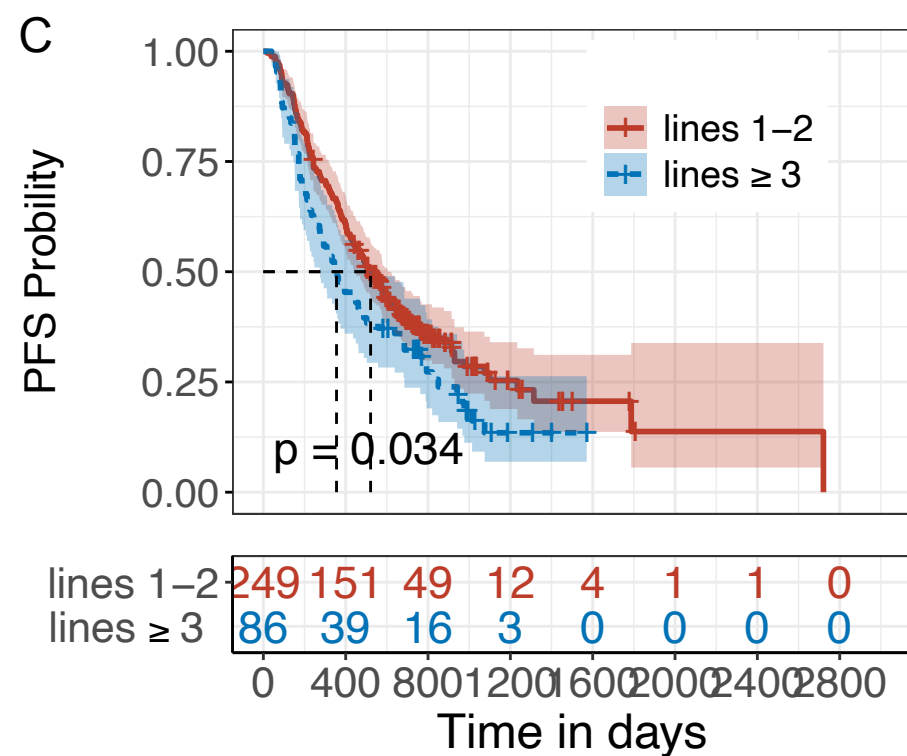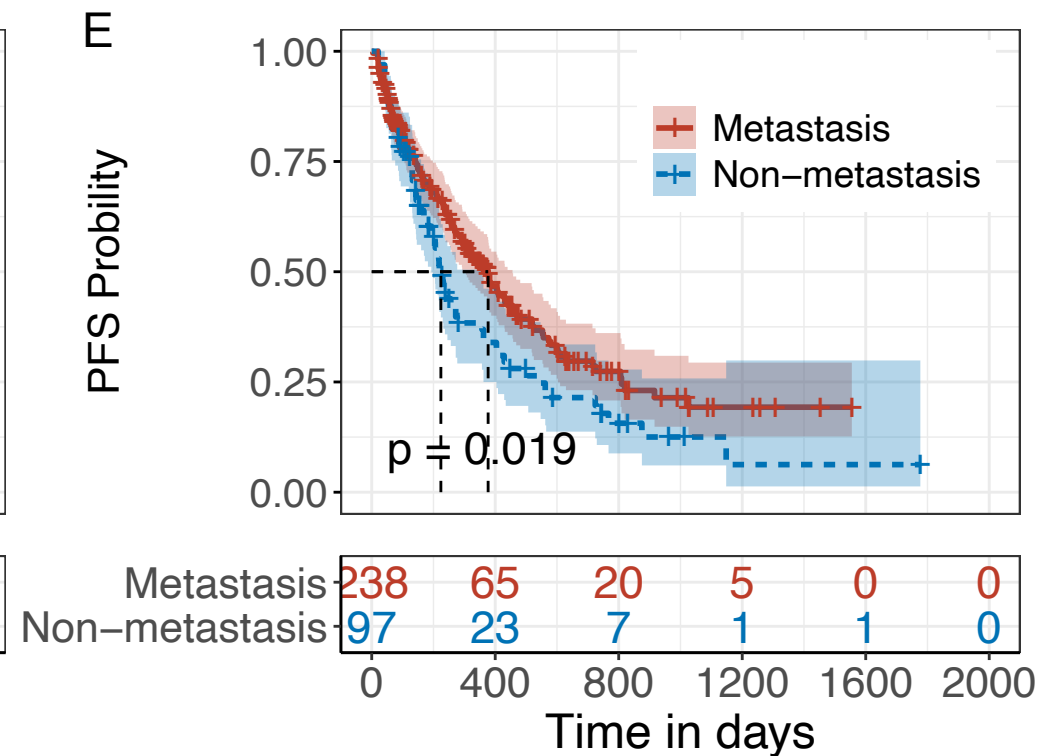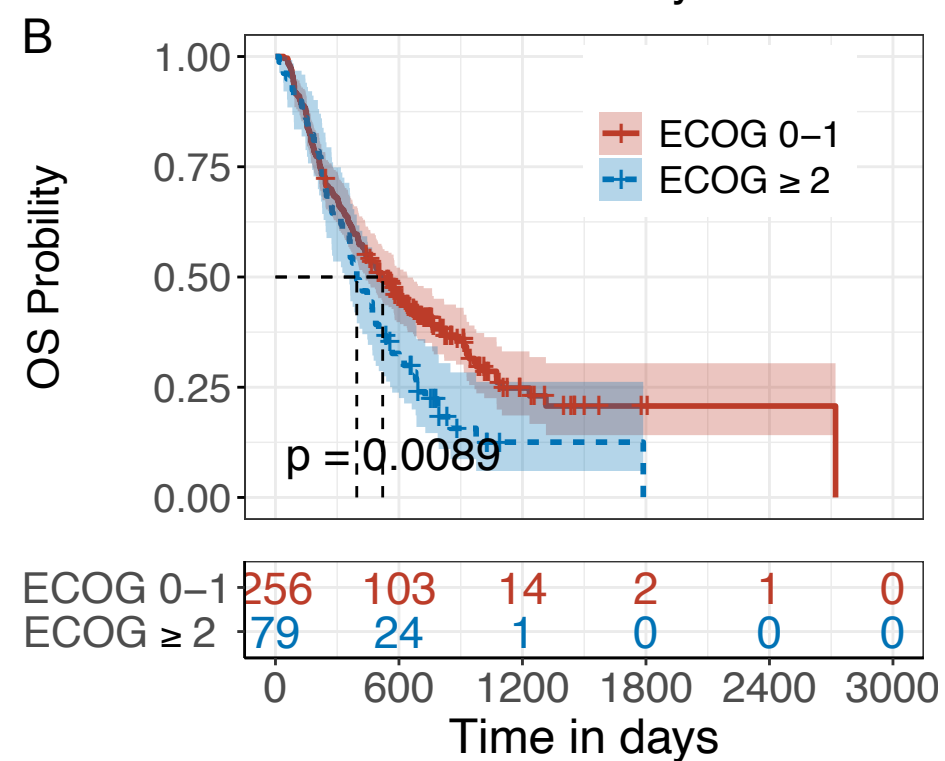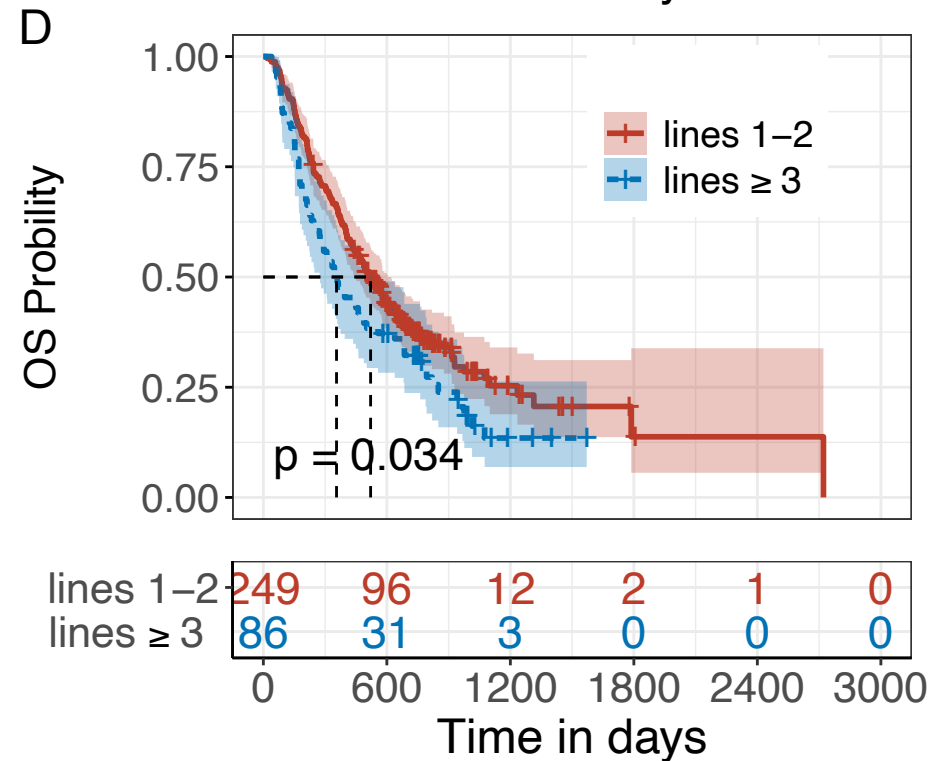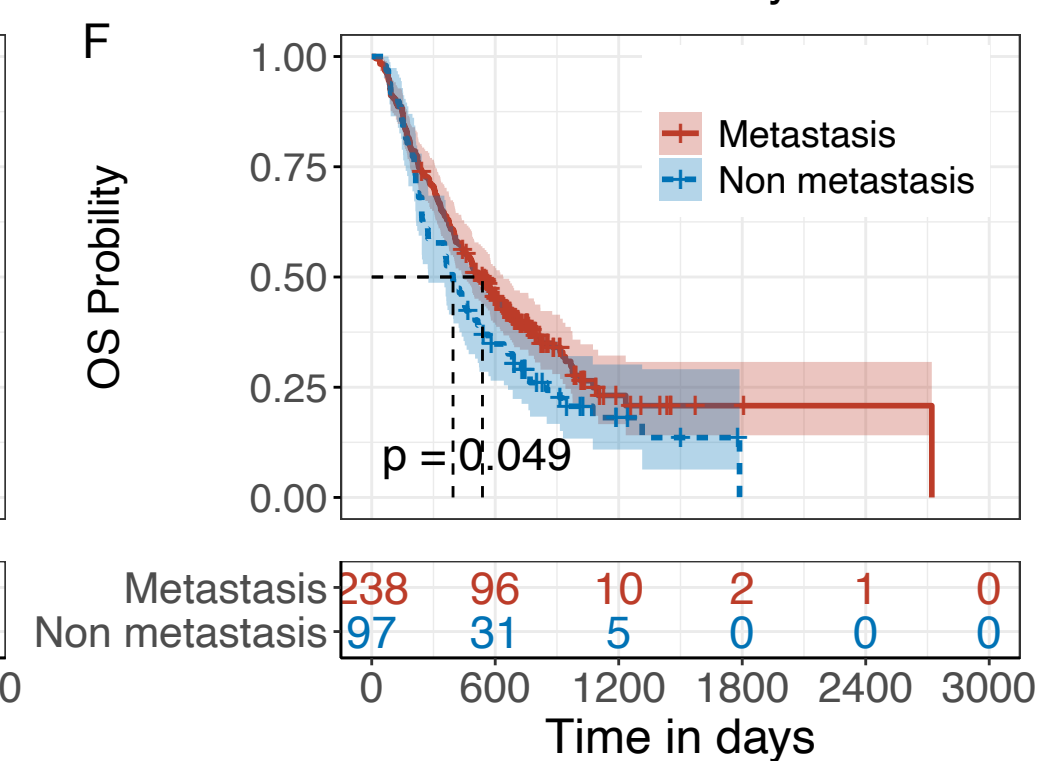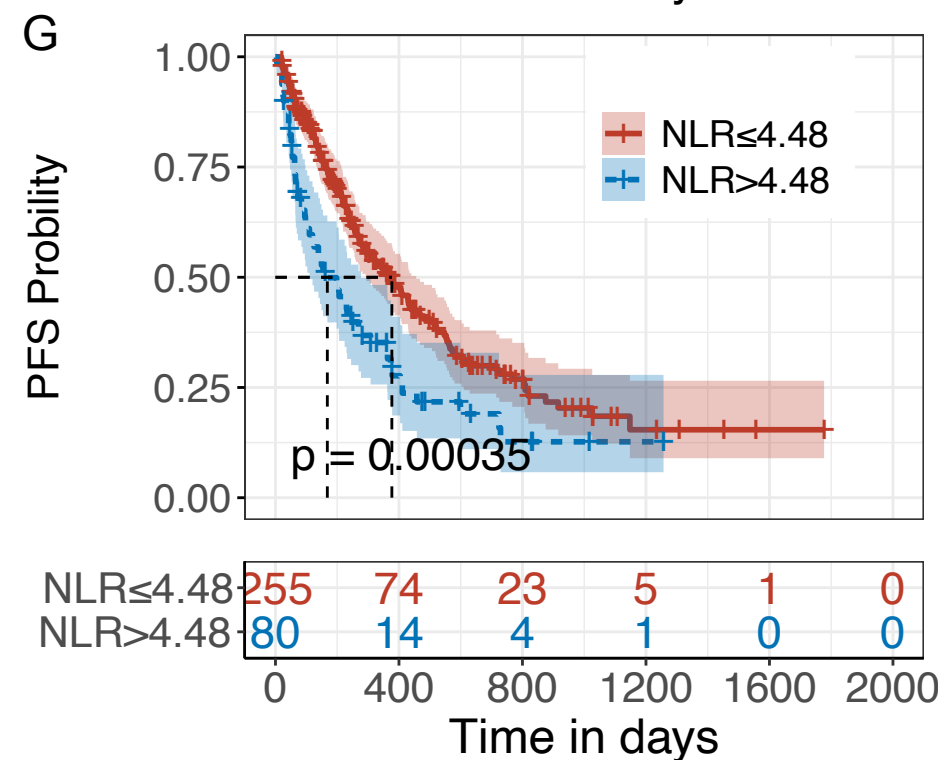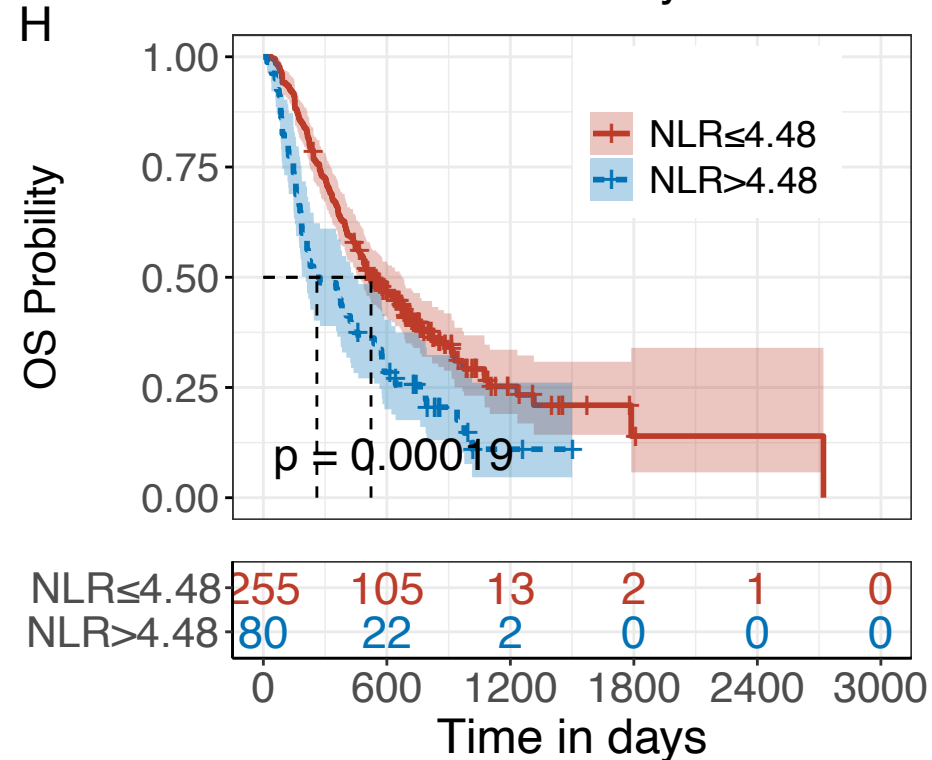

Supplement: Supplementary file 3 — Supporting File 3 [file IID3-14-e70402-s003.pdf]

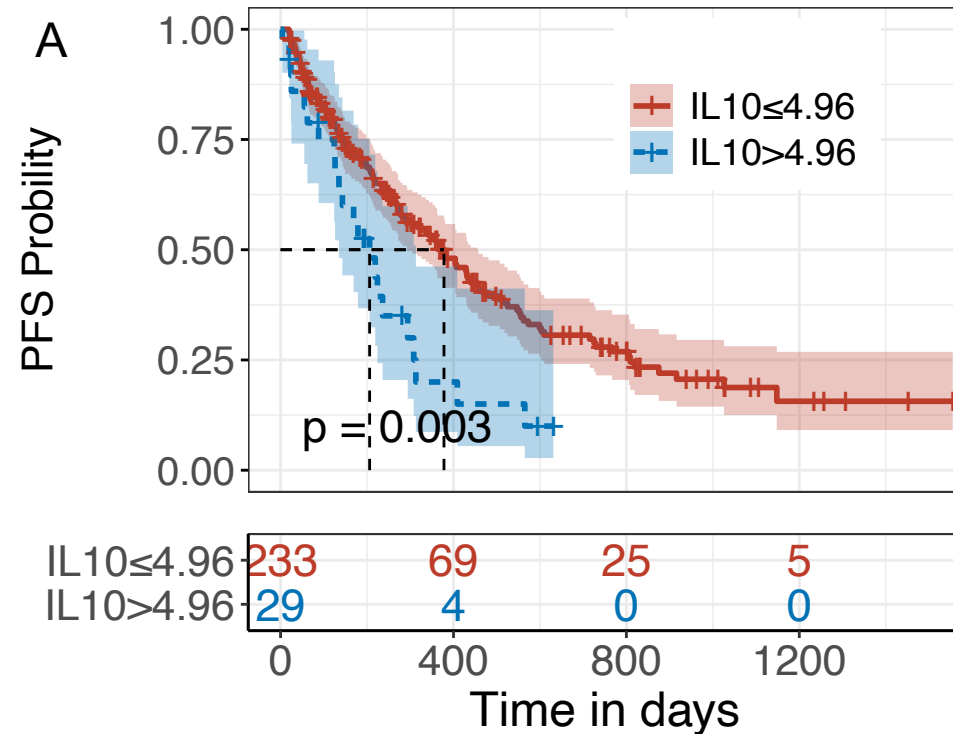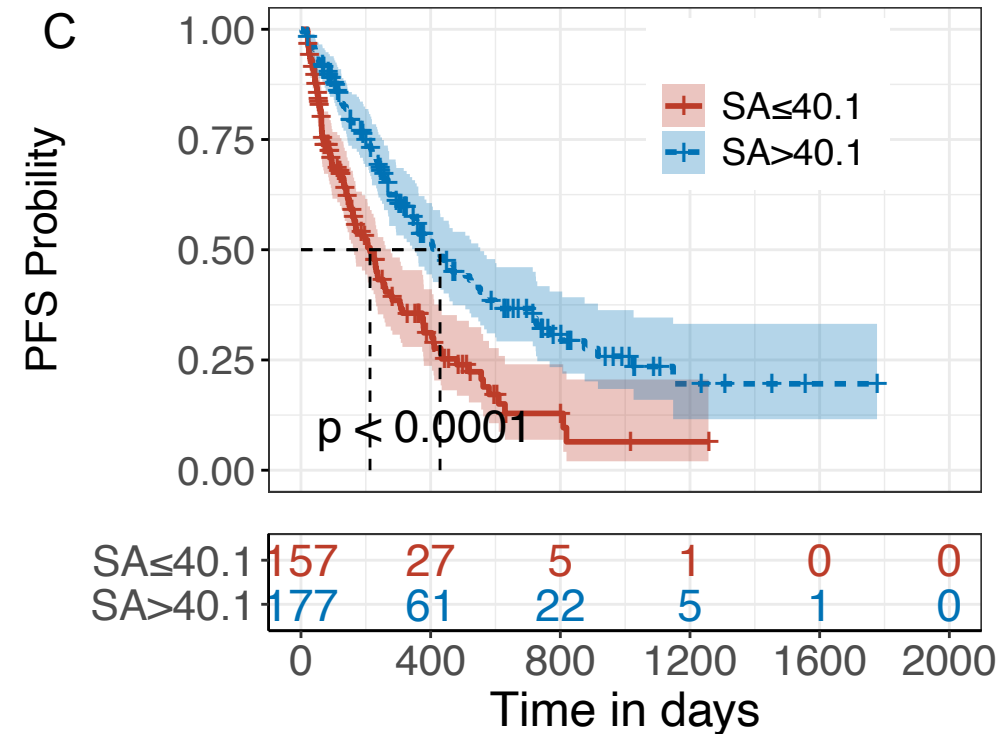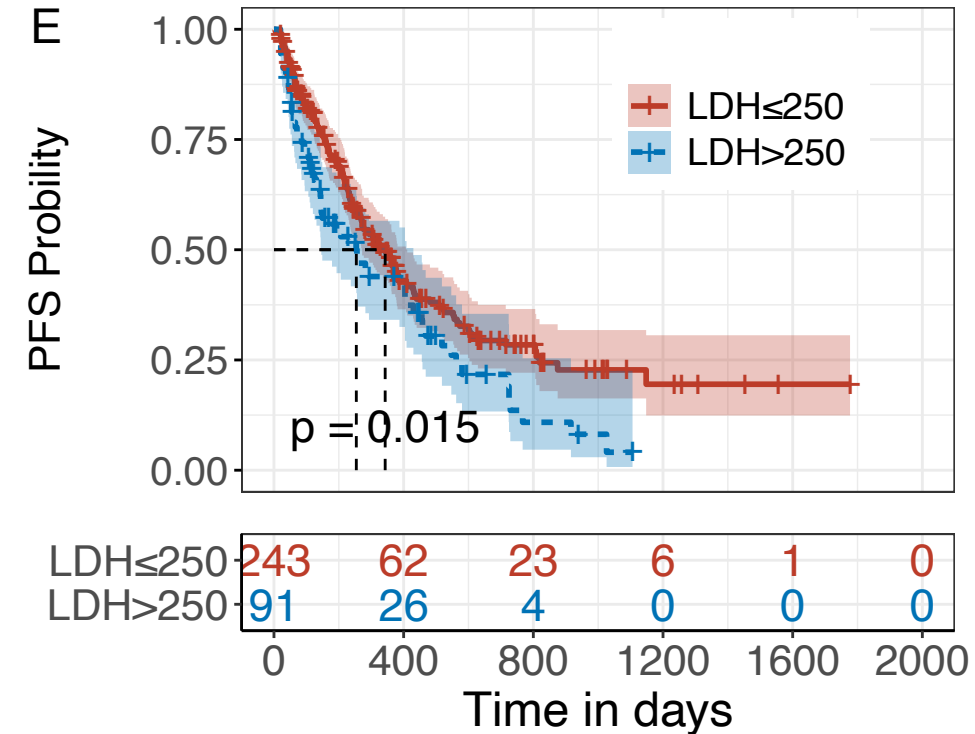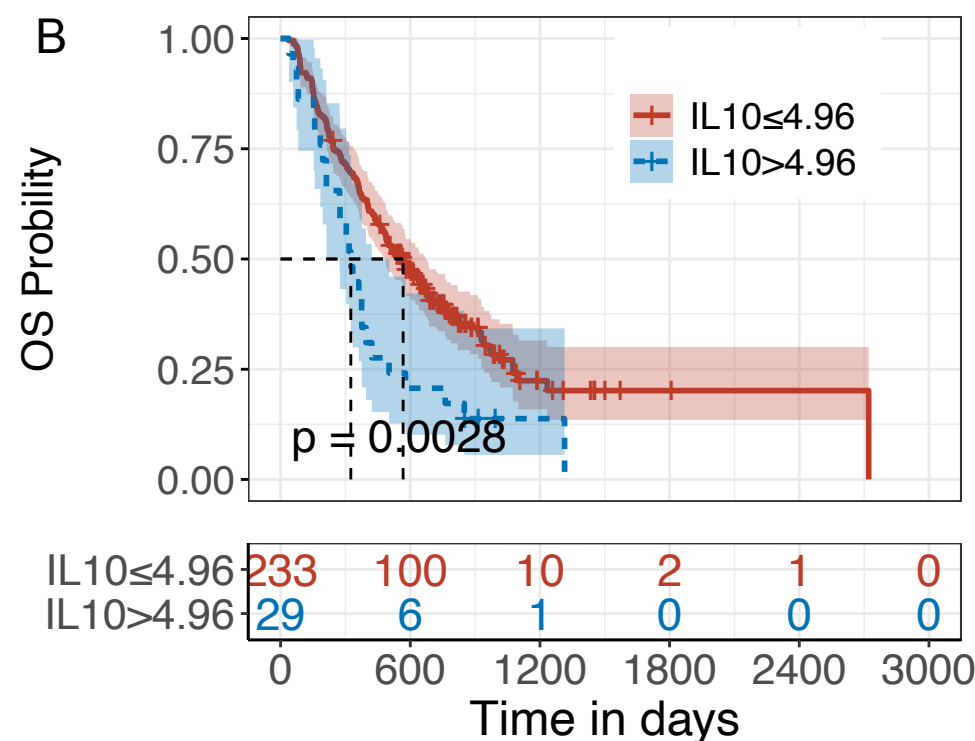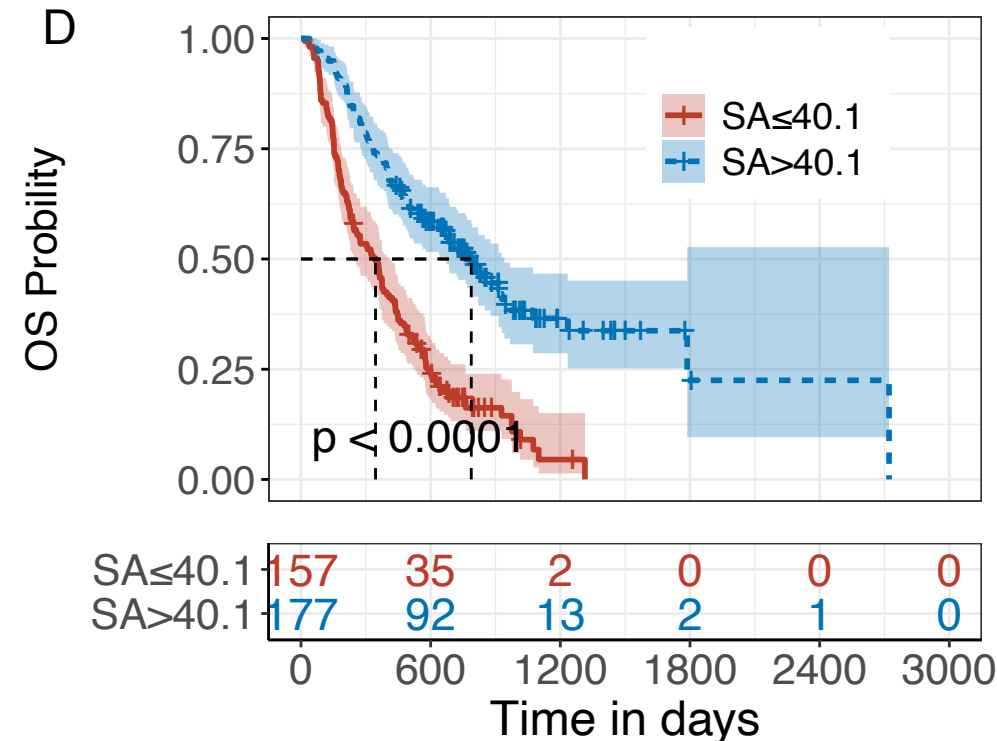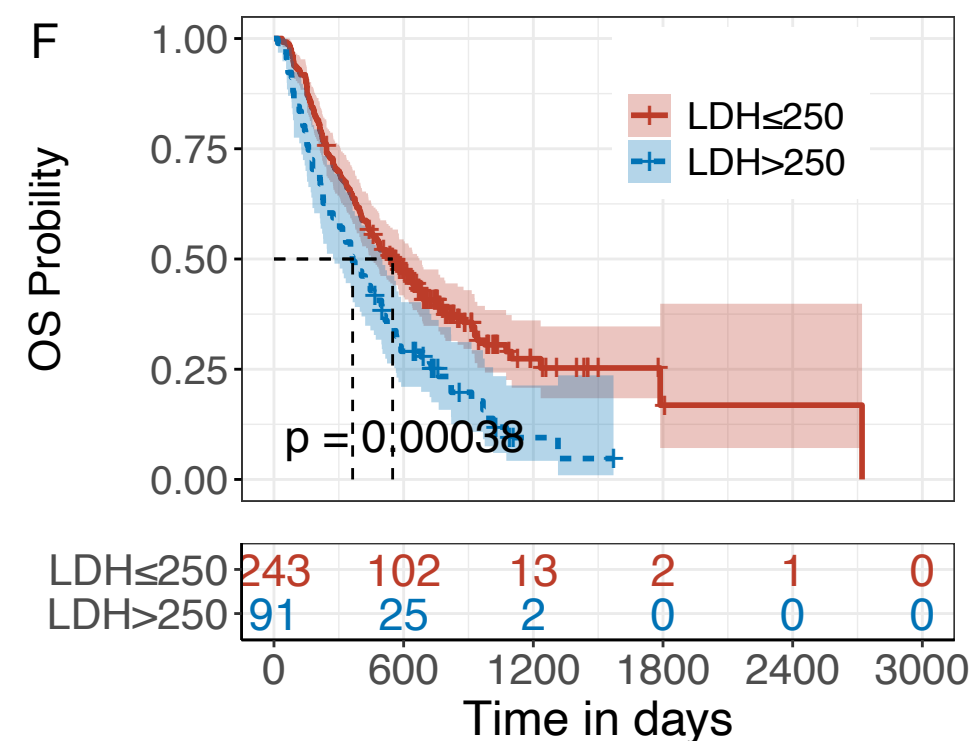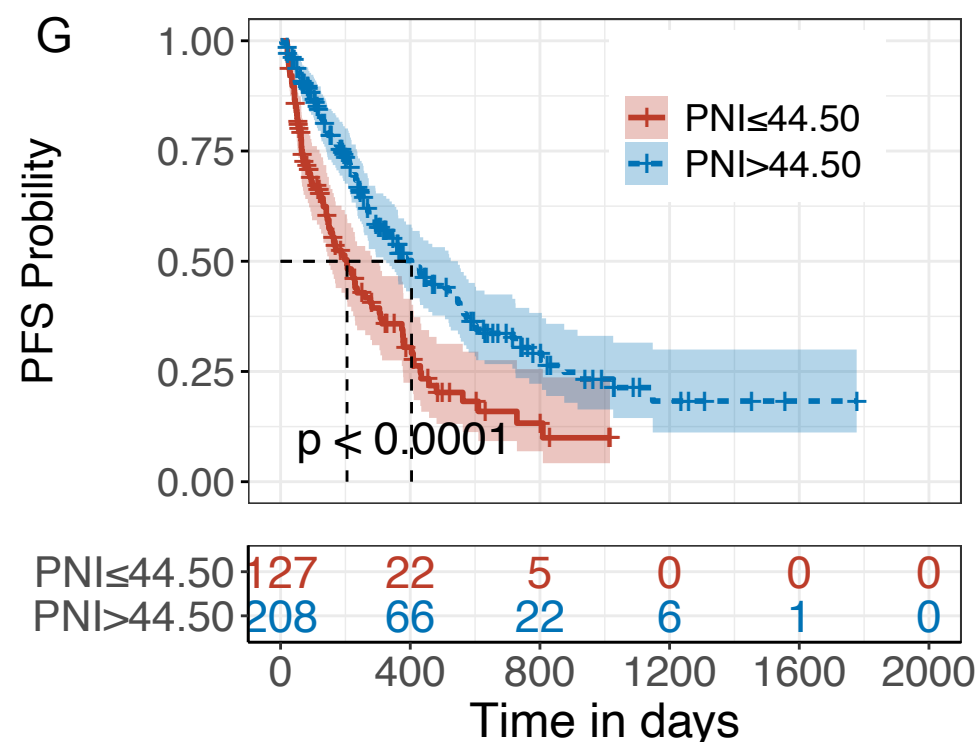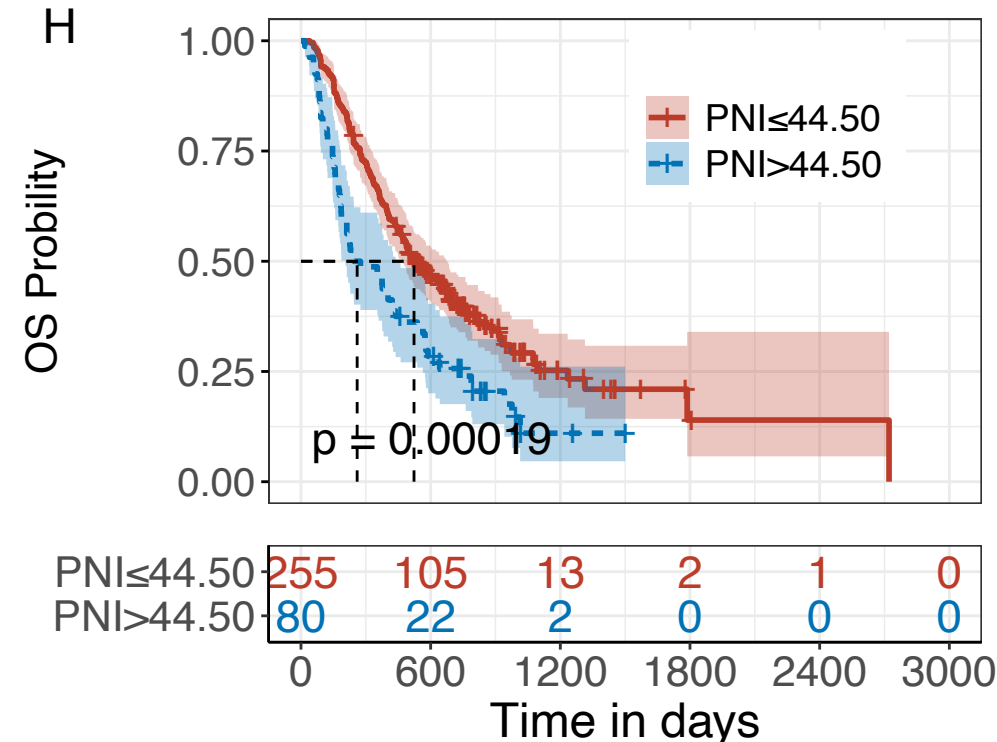

Supplement: Supplementary file 4 — Supporting File 4 [file IID3-14-e70402-s008.pdf]

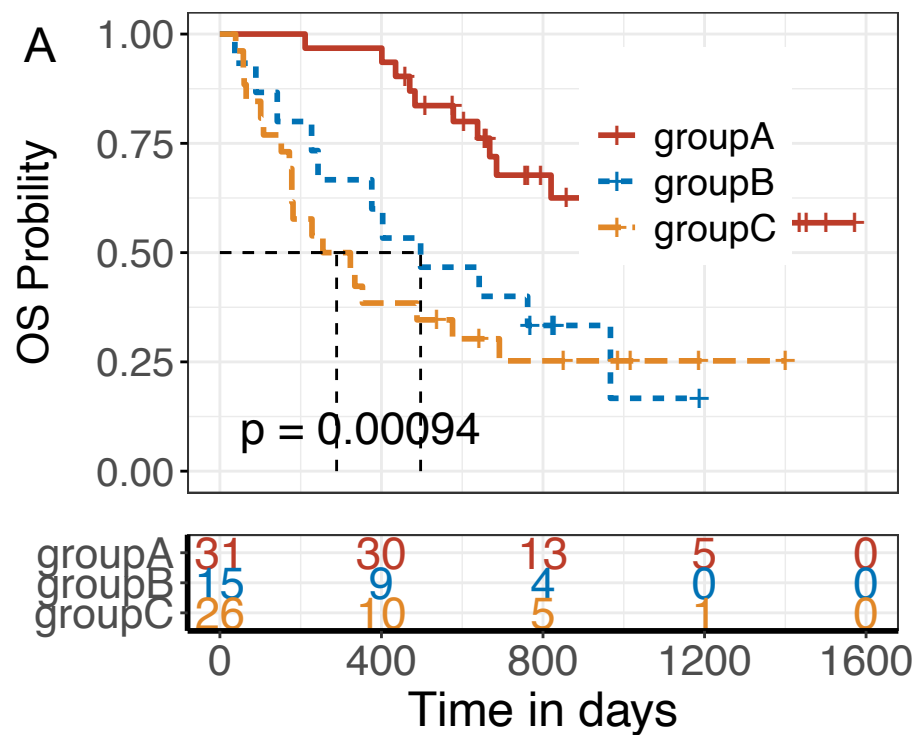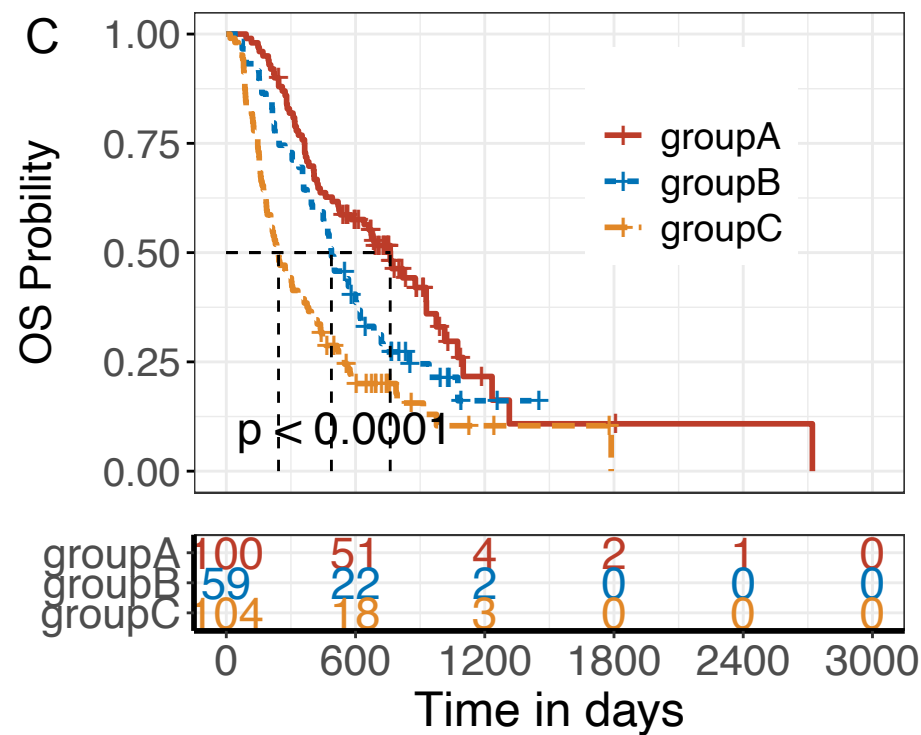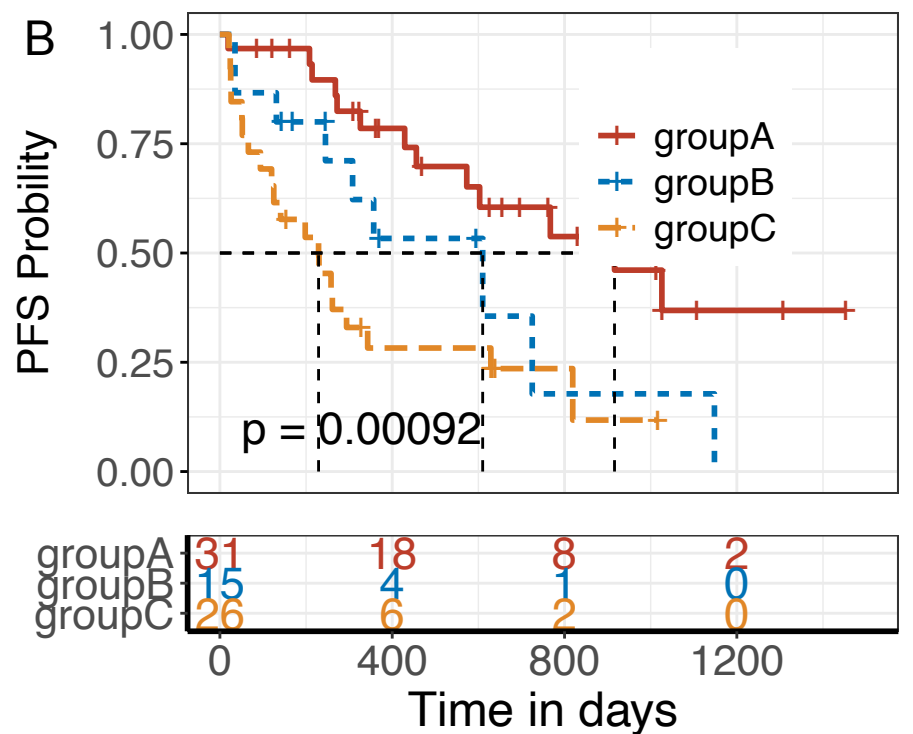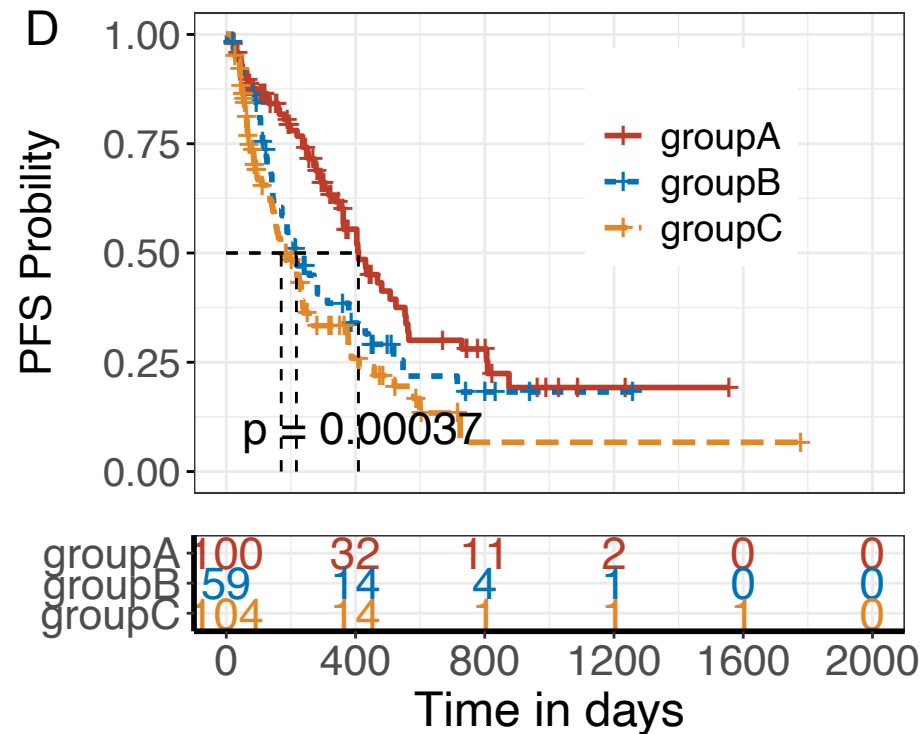

Supplement: Supplementary file 5 — Supporting File 5 [file IID3-14-e70402-s009.pdf]

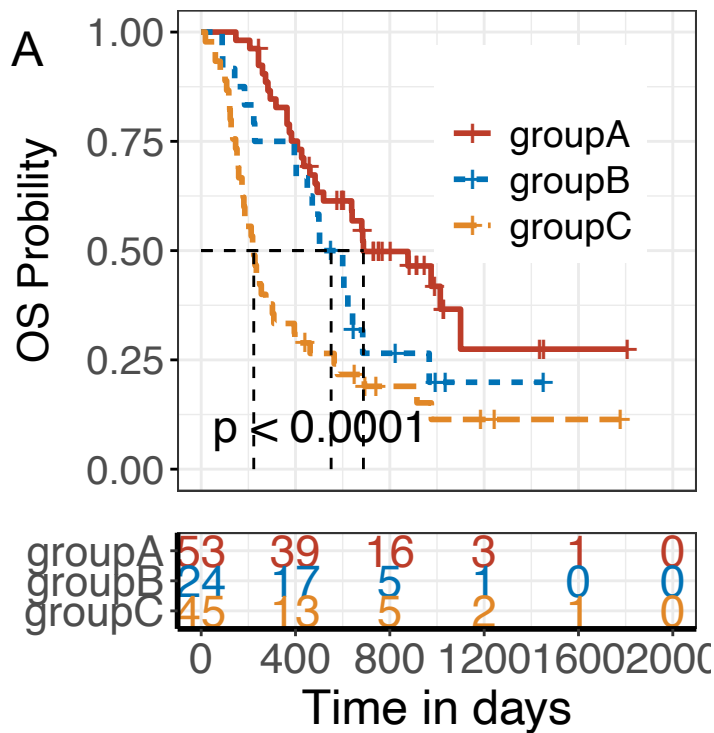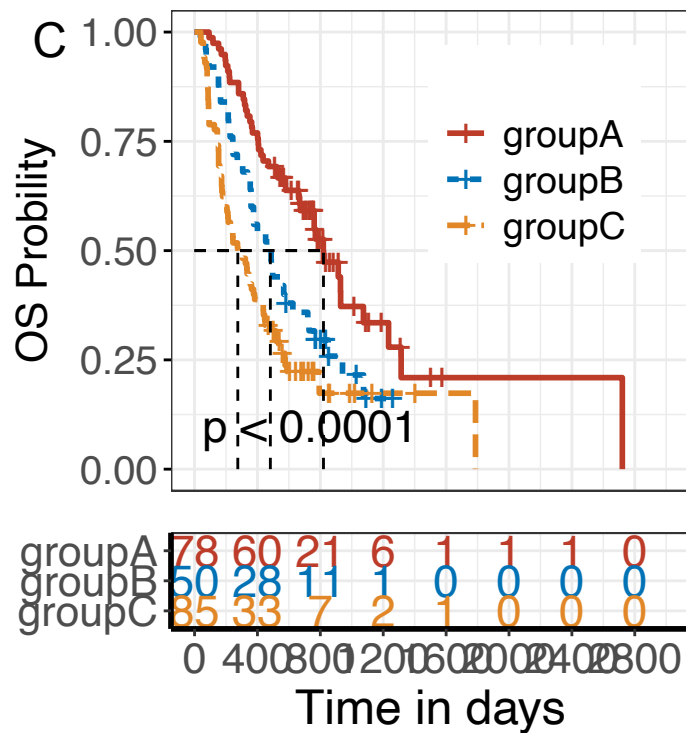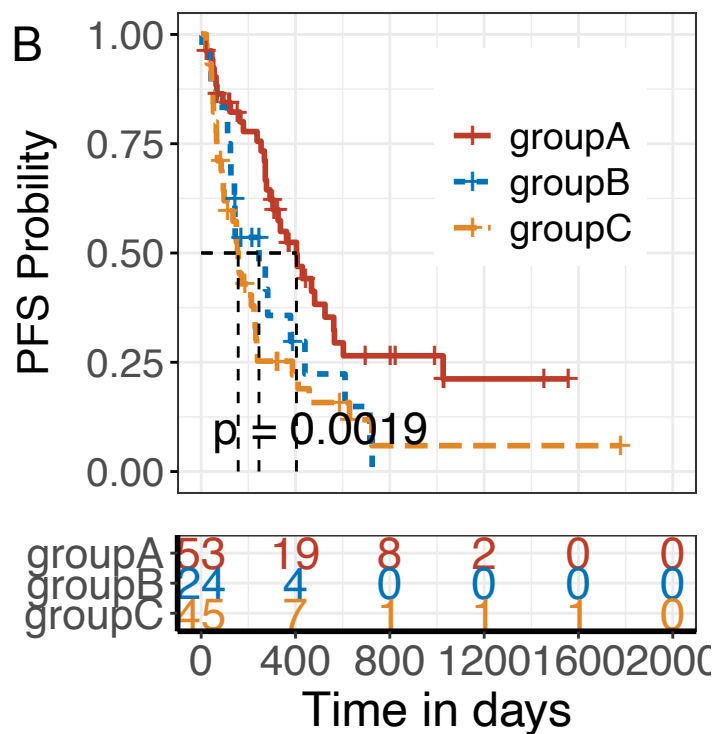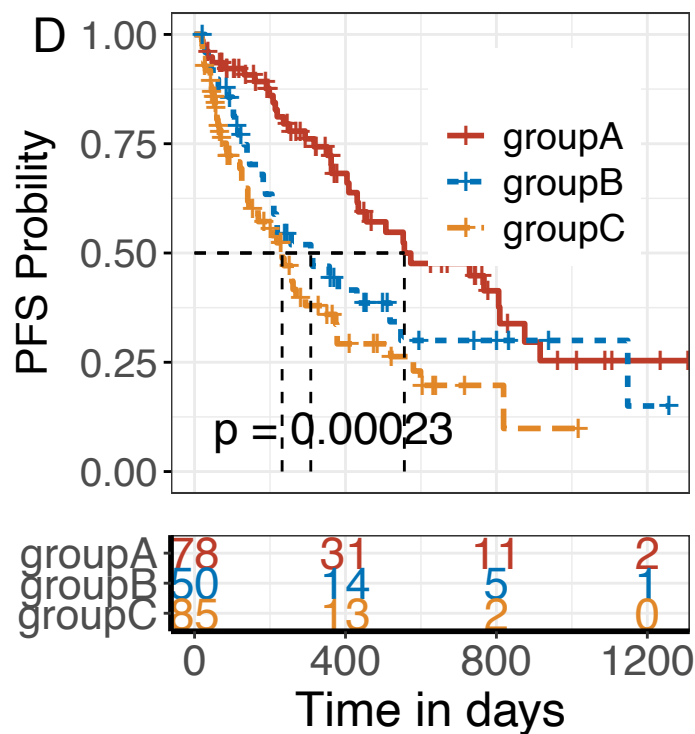

Supplement: Supplementary file 6 — Supporting File 6 [file IID3-14-e70402-s015.pdf]

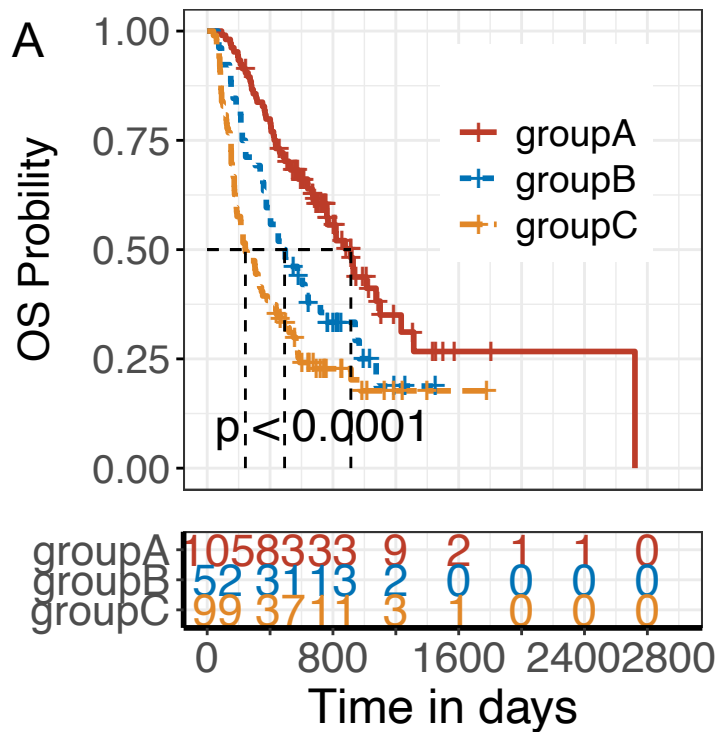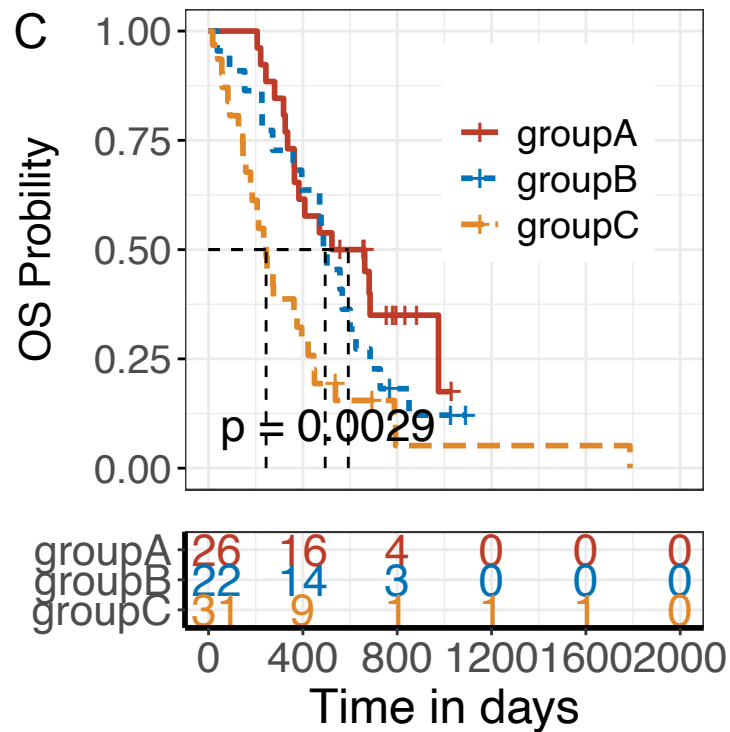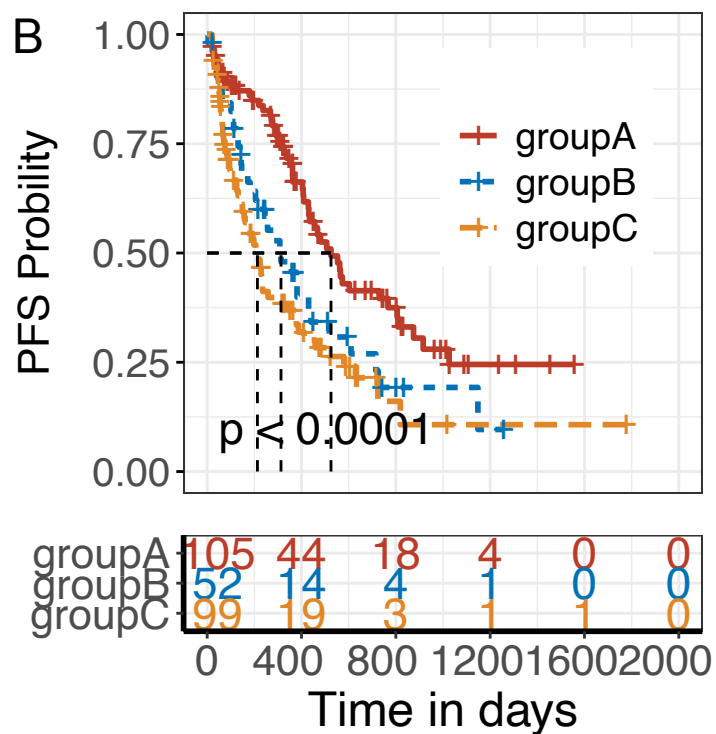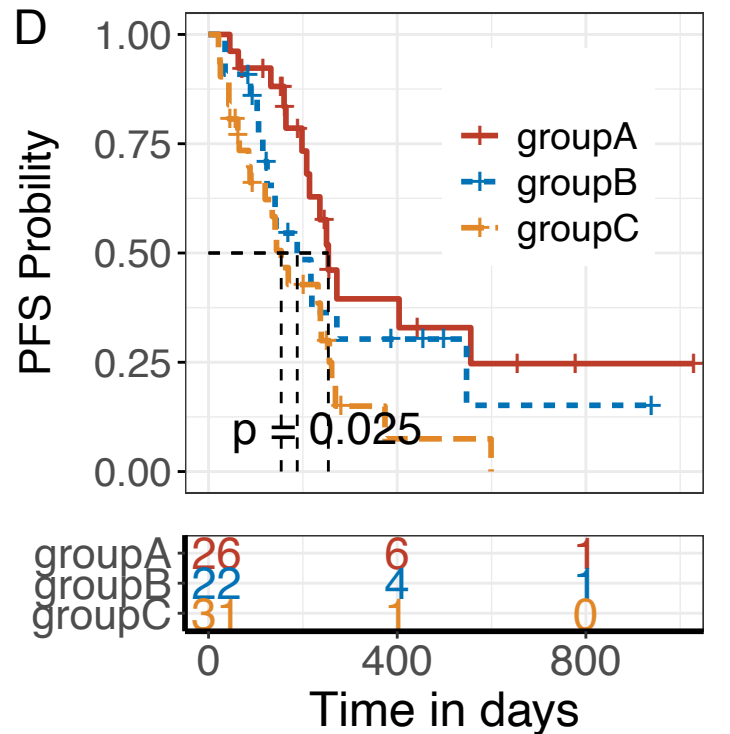

Supplement: Supplementary file 7 — Supporting File 7 [file IID3-14-e70402-s013.pdf]

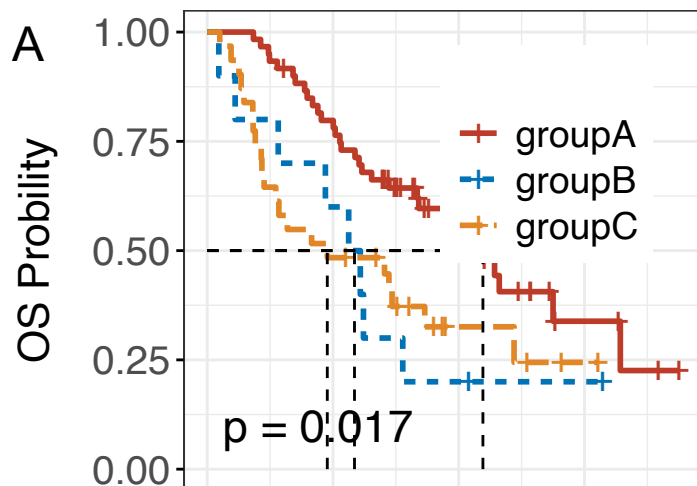

|        |    |    |    |   |
|--------|----|----|----|---|
| groupA | 60 | 47 | 19 | 4 |
| groupB | 10 | 6  | 2  | 1 |
| groupC | 31 | 15 | 4  | 1 |

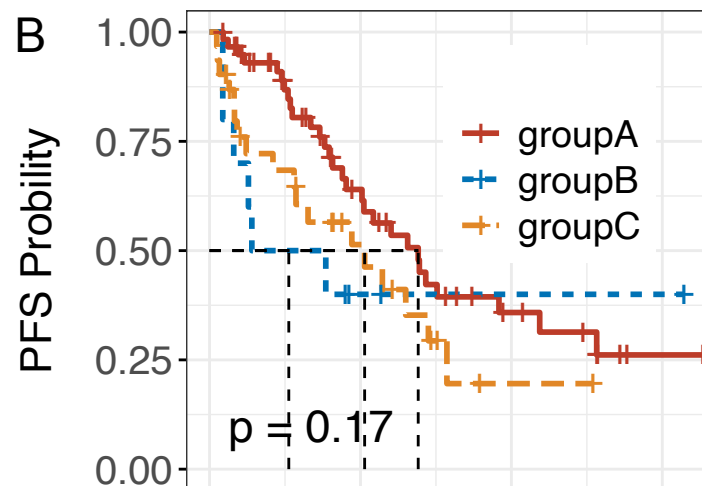

|        |    |    |   |   |
|--------|----|----|---|---|
| groupA | 60 | 25 | 9 | 1 |
| groupB | 10 | 2  | 1 | 1 |
| groupC | 31 | 10 | 1 | 0 |

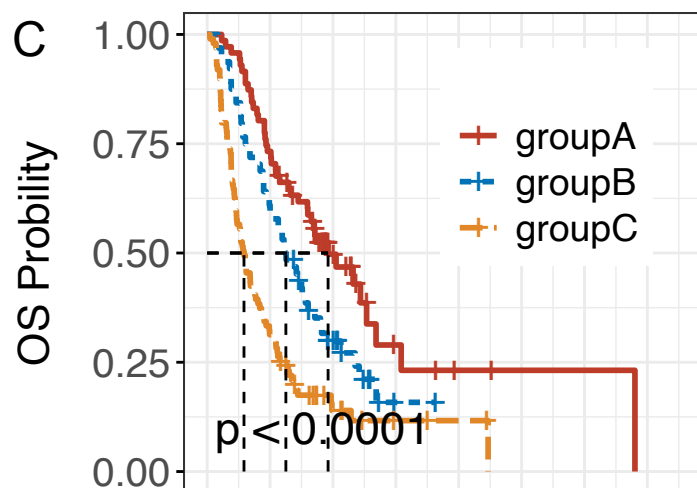

|        |    |    |    |   |   |   |   |   |
|--------|----|----|----|---|---|---|---|---|
| groupA | 71 | 52 | 18 | 5 | 2 | 1 | 1 | 0 |
| groupB | 64 | 39 | 14 | 1 | 0 | 0 | 0 | 0 |
| groupC | 99 | 31 | 8  | 3 | 2 | 0 | 0 | 0 |

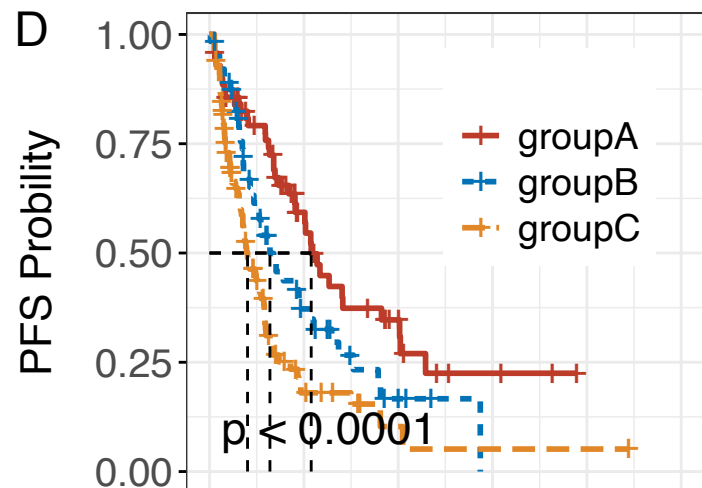

|        |    |    |    |   |   |   |
|--------|----|----|----|---|---|---|
| groupA | 71 | 25 | 10 | 3 | 0 | 0 |
| groupB | 64 | 16 | 4  | 0 | 0 | 0 |
| groupC | 99 | 10 | 2  | 1 | 1 | 0 |

Supplement: Supplementary file 8 — Supporting File 8 [file IID3-14-e70402-s007.pdf]

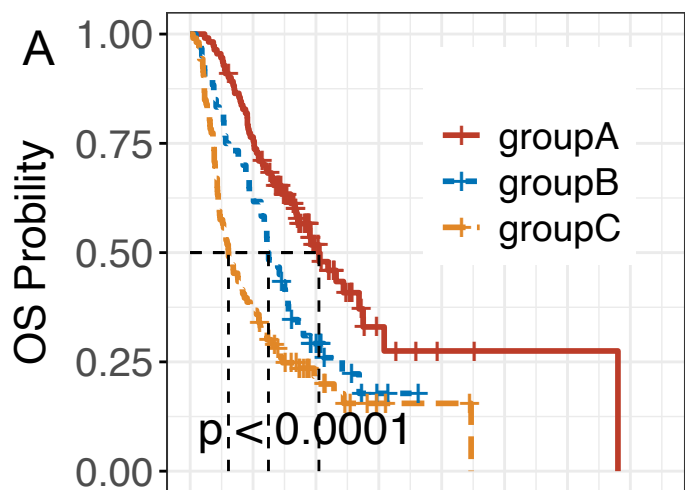

|        |   |   |    |    |    |   |   |   |   |   |
|--------|---|---|----|----|----|---|---|---|---|---|
| groupA | 1 | 1 | 18 | 4  | 28 | 6 | 2 | 1 | 1 | 0 |
| groupB | 6 | 0 | 38 | 13 | 2  | 0 | 0 | 0 | 0 | 0 |
| groupC | 1 | 0 | 6  | 39 | 11 | 3 | 2 | 0 | 0 | 0 |

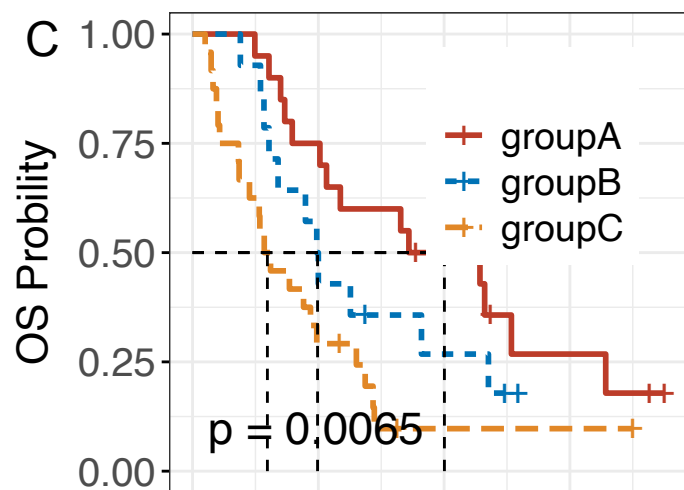

|        |    |    |   |   |
|--------|----|----|---|---|
| groupA | 20 | 15 | 9 | 3 |
| groupB | 14 | 7  | 3 | 0 |
| groupC | 24 | 7  | 1 | 1 |

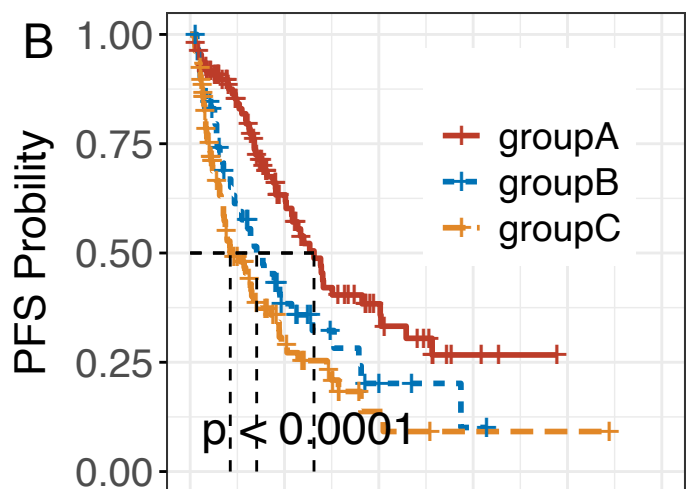

|        |   |   |    |    |    |   |   |   |
|--------|---|---|----|----|----|---|---|---|
| groupA | 1 | 1 | 4  | 1  | 15 | 3 | 0 | 0 |
| groupB | 6 | 0 | 15 | 4  | 1  | 0 | 0 | 0 |
| groupC | 1 | 0 | 6  | 17 | 3  | 1 | 1 | 0 |

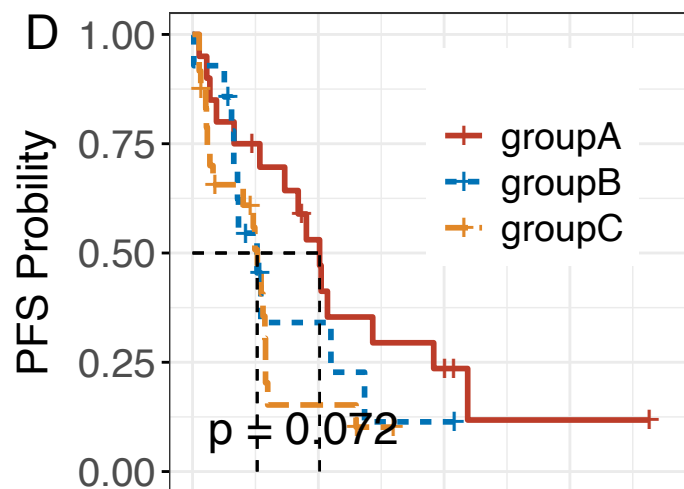

|        |    |   |   |   |
|--------|----|---|---|---|
| groupA | 20 | 9 | 4 | 1 |
| groupB | 14 | 3 | 1 | 0 |
| groupC | 24 | 3 | 0 | 0 |

Supplement: Supplementary file 9 — Supporting File 9 [file IID3-14-e70402-s010.pdf]

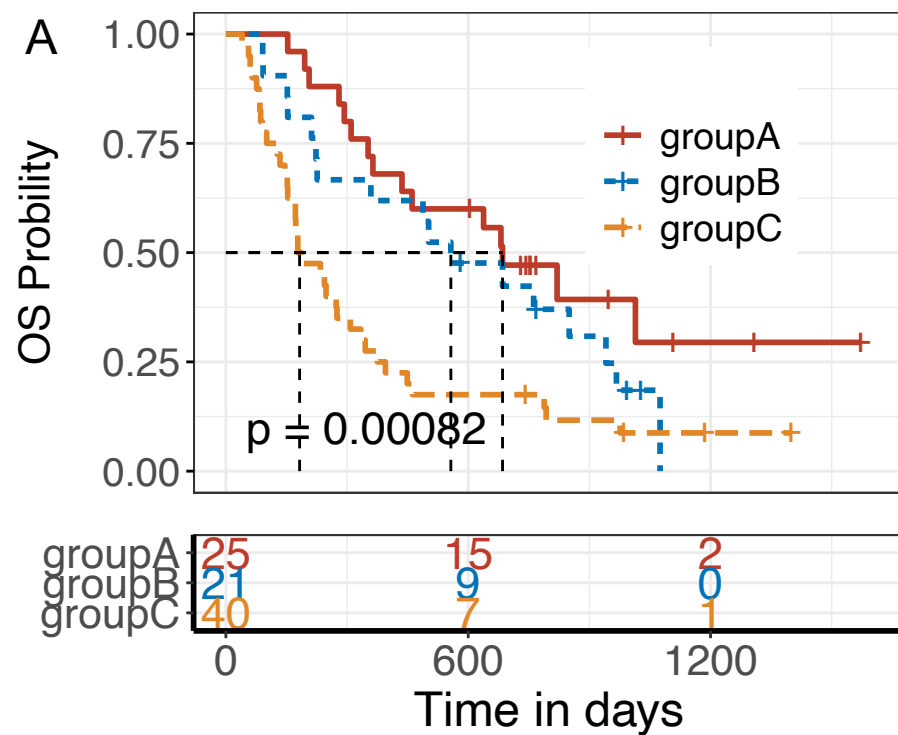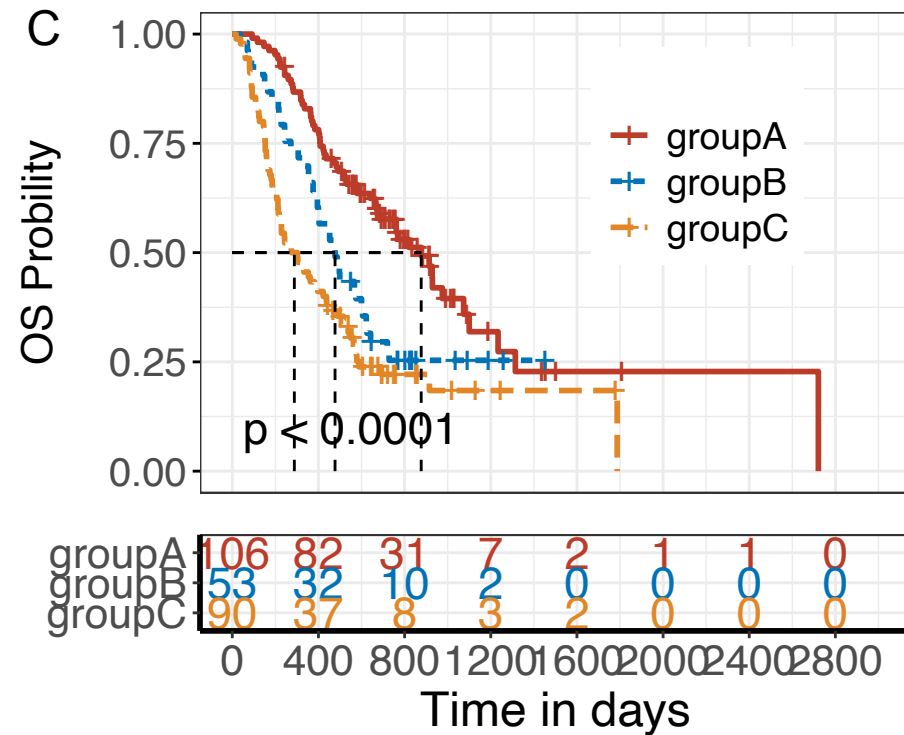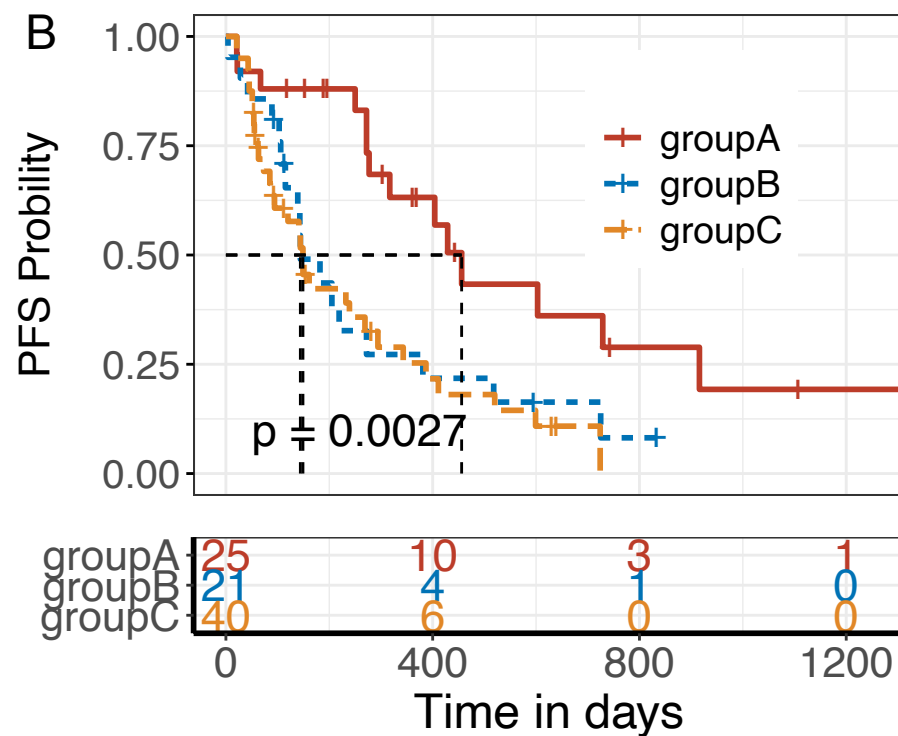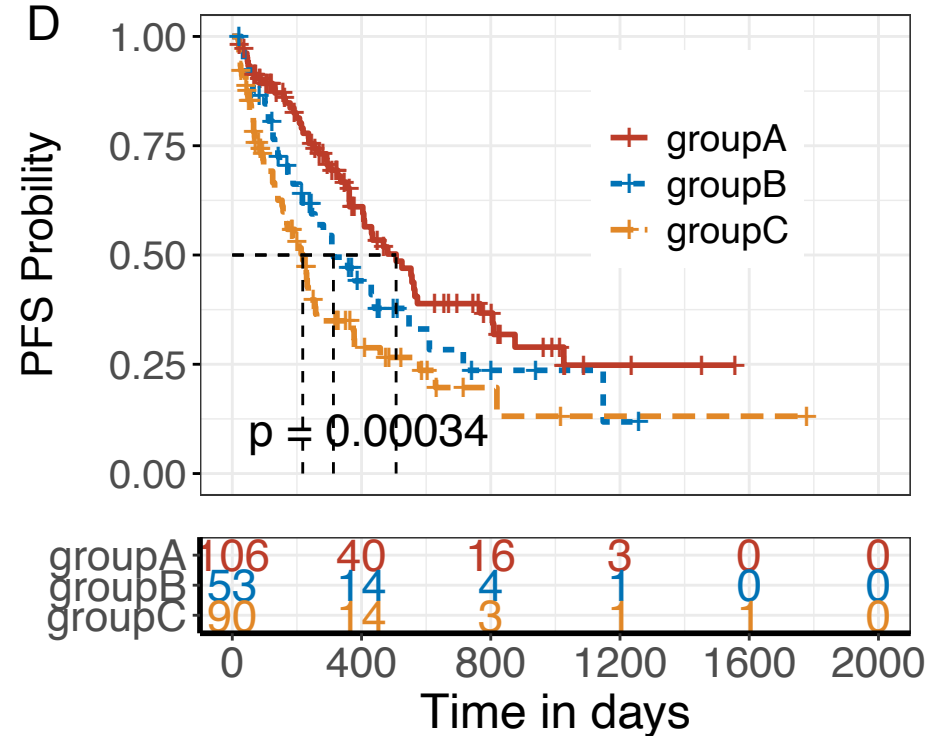

Supplement: Supplementary file 10 — Supporting File 10 [file IID3-14-e70402-s006.pdf]

Variable

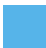

groupA

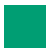

groupB

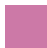

groupC

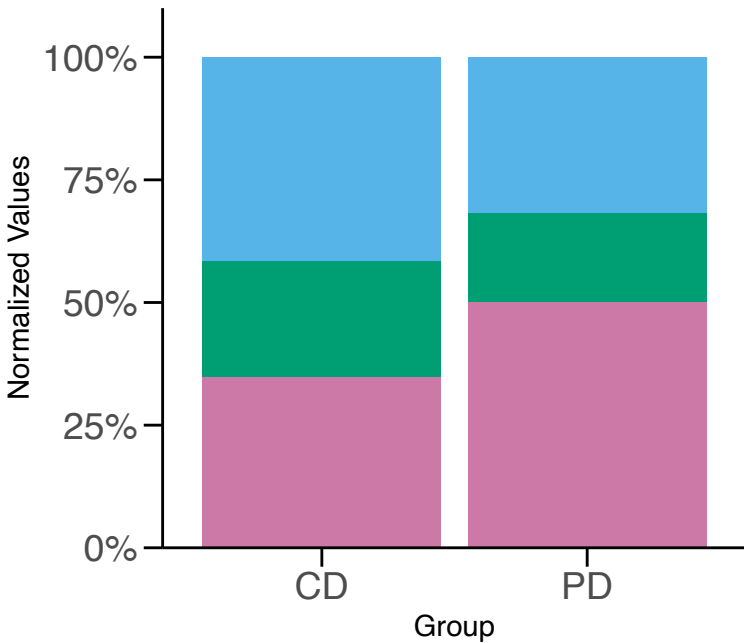

Supplement: Supplementary file 11 — Supporting File 11 [file IID3-14-e70402-s002.pdf]

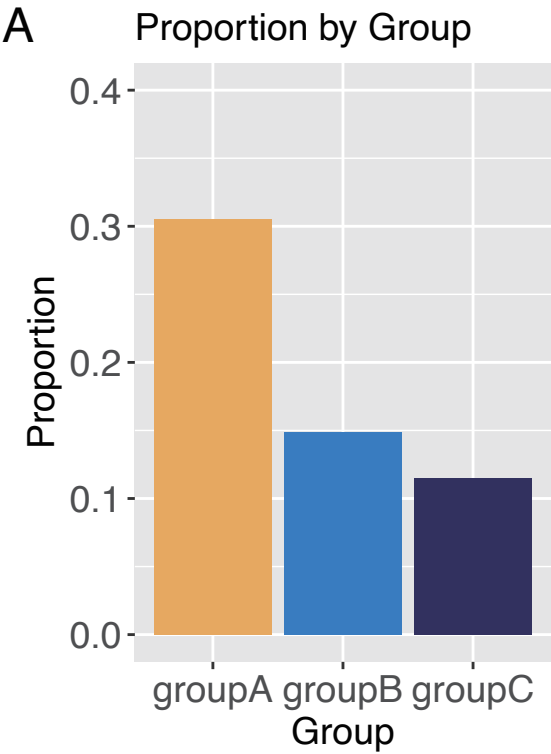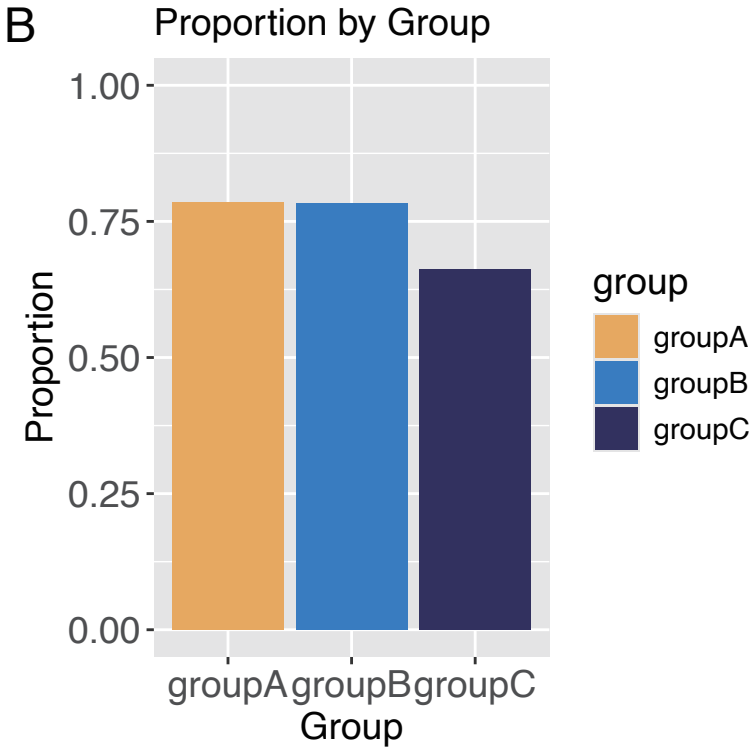

Supplement: Supplementary file 12 — Supporting File 12 [file IID3-14-e70402-s011.pdf]

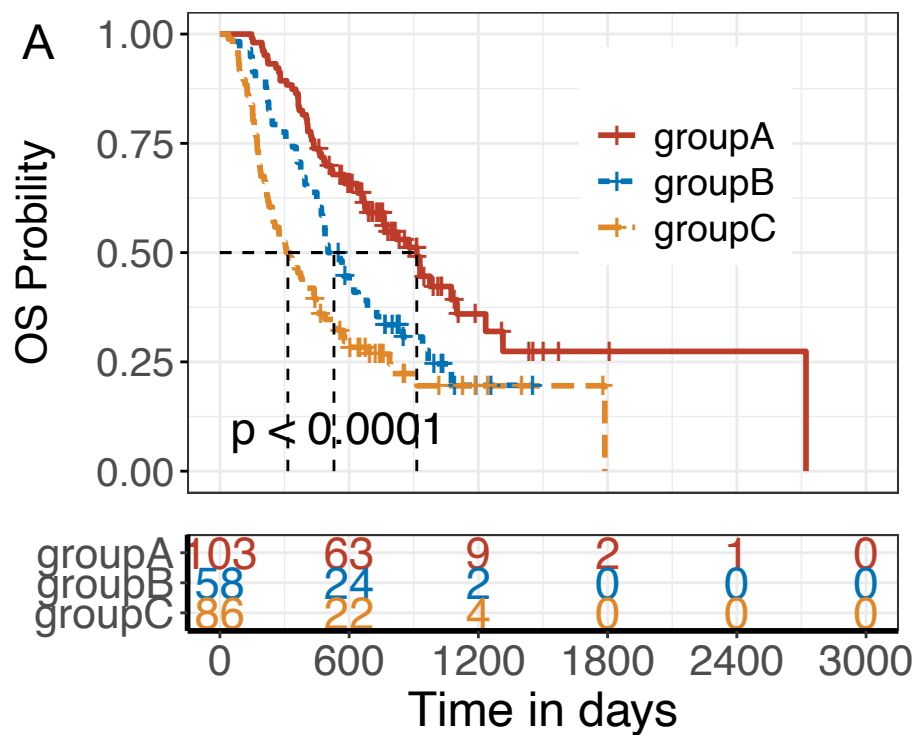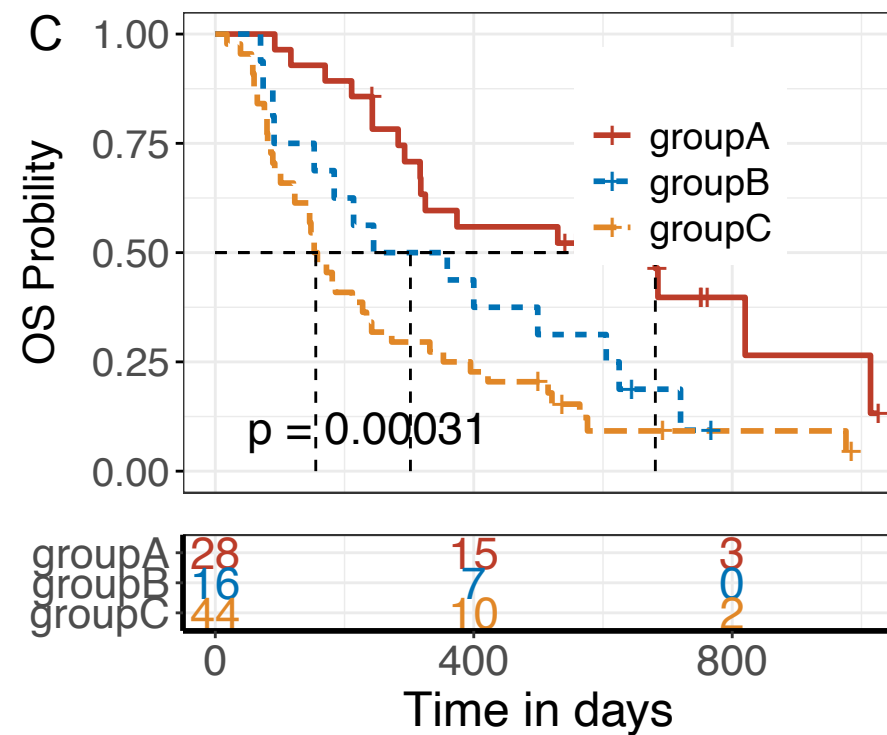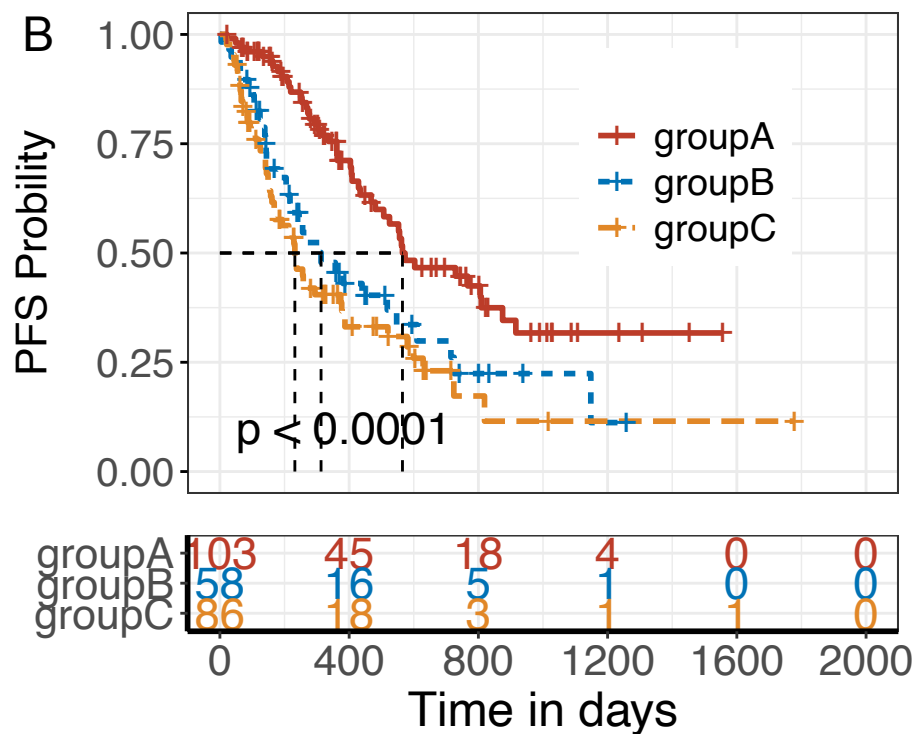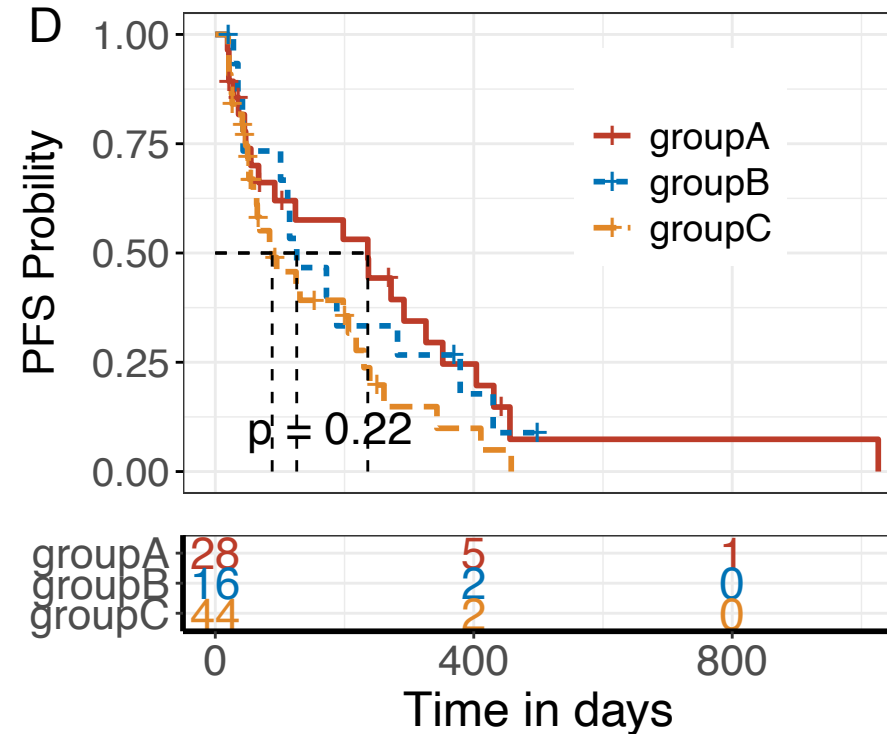

Supplement: Supplementary file 13 — Supporting File 13 [file IID3-14-e70402-s001.pdf]
