# Supplementary material for: Peripheral Blood Biomarkers Predict Outcomes in Advanced Cancers Treated With Anti‐PD‐1 Therapy
Source: Immun Inflamm Dis. 2026 May 6;14(5):e70402. doi: 10.1002/iid3.70402 (PMC13149763; doi:10.1002/iid3.70402)
Supplement: Supplementary file 14 — Supporting File 14 [file IID3-14-e70402-s012.docx]

Supplementary Figure Legends

Supplementary Fig. 1 Kaplan-Meier analysis of overall survival (OS) and progression-free survival (PFS) stratified by radiological response.

(A) Kaplan-Meier curves illustrate the probability of OS. (B) Kaplan-Meier curves represent the probability of PFS. CD group, Clinically Beneficial Disease group; PD group, Progressive Disease group.

Supplementary Fig. 2 Kaplan-Meier analysis of overall survival (OS) and progression-free survival (PFS) stratified by pathological type and peripheral blood biomarker (NLR)

(A, B) Kaplan-Meier curves based on the Eastern Cooperative Oncology Group (ECOG) performance status cutoff value. (C, D) Kaplan-Meier curves based on the treatment lines cutoff value. (E, F) Kaplan-Meier curves based on metastasis status. (G, H) Survival curves based on NLR cutoff values. *P*-values were computed using the log-rank test.

Supplementary Fig. 3 Kaplan-Meier analysis of overall survival (OS) and progression-free survival (PFS) stratified by peripheral blood biomarkers

(A, B) Kaplan-Meier curves based on IL10 cutoff value. (C, D) Kaplan-Meier curves based on the serum albumin (SA) cutoff value . (E, F) Kaplan-Meier curves based on LDH cutoff value. (G, H) Survival curves based on PNI cutoff values. *P*-values were computed using the log-rank test.

Supplementary Fig. 4 Kaplan-Meier curves depicting overall survival (OS) and progression-free survival (PFS) according to the number of favorable factors present at baseline. Factors include squamous cell carcinoma histology, dNLR ≤ 3.18, AMC ≤ 0.48, and IL6 ≤ 12.35. Patient groups are categorized as follows: Group A (with ≥ three factors), Group B (with two factors), and Group C (with ≤ one factor)

(A, B) Represent the survival outcomes for the immunotherapy group (n=72) based on the multifactorial model. (C, D) Show the subgroup analysis of the multifactorial model within the immunotherapy combination treatment group (n=263). *P*-Values were determined using the log-rank test.

Supplementary Fig. 5 Kaplan-Meier curves depicting overall survival (OS) and progression-free survival (PFS) according to the number of favorable factors present at baseline. Factors include squamous cell carcinoma histology, dNLR ≤ 3.18, AMC ≤ 0.48, and IL6 ≤ 12.35. Patient groups are categorized as follows: Group A (with ≥ three factors), Group B (with two factors), and Group C (with ≤ one factor)

(A, B) Represent the survival outcomes in woman patients (n=122) based on the multifactorial model. (C, D) Show the subgroup analysis of the multifactorial model in men (n=263). *P*-Values were determined using the log-rank test.

Supplementary Fig. 6 Kaplan-Meier curves depicting overall survival (OS) and progression-free survival (PFS) according to the number of favorable factors present at baseline. Factors include squamous cell carcinoma histology, dNLR ≤ 3.18, AMC ≤ 0.48, and IL6 ≤ 12.35. Patient groups are categorized as follows: Group A (with ≥ three factors), Group B (with two factors), and Group C (with ≤ one factor)

(A, B) Represent the survival outcomes in ECOG PS scores 0-1 group (n=256) based on the multifactorial model. (C, D) Show the subgroup analysis of the multifactorial model in ECOG PS scores ≥ 2 (n=79). *P*-Values were determined using the log-rank test.

Supplementary Fig. 7 Kaplan-Meier curves depicting overall survival (OS) and progression-free survival (PFS) according to the number of favorable factors present at baseline. Factors include squamous cell carcinoma histology, dNLR ≤ 3.18, AMC ≤ 0.48, and IL6 ≤ 12.35. Patient groups are categorized as follows: Group A (with ≥ three factors), Group B (with two factors), and Group C (with ≤ one factor)

(A, B) Represent the survival outcomes in squamous carcinoma group (n=101) based on the multifactorial model. (C, D) Show the subgroup analysis of the multifactorial model in non-squamous carcinoma group (n=234). *P*-Values were determined using the log-rank test.

Supplementary Fig. 8 Kaplan-Meier curves depicting overall survival (OS) and progression-free survival (PFS) according to the number of favorable factors present at baseline. Factors include squamous cell carcinoma histology, dNLR ≤ 3.18, AMC ≤ 0.48, and IL6 ≤ 12.35. Patient groups are categorized as follows: Group A (with ≥ three factors), Group B (with two factors), and Group C (with ≤ one factor)

(A, B) Represent the survival outcomes in number of metastatic sites 0-1 group (n=277) based on the multifactorial model. (C, D) Show the subgroup analysis of the multifactorial model in number of metastatic sites ≥3 group (n=58). *P*-Values were determined using the log-rank test.

Supplementary Fig. 9 Kaplan-Meier curves depicting overall survival (OS) and progression-free survival (PFS) according to the number of favorable factors present at baseline. Factors include squamous cell carcinoma histology, dNLR ≤ 3.18, AMC ≤ 0.48, and IL6 ≤ 12.35. Patient groups are categorized as follows: Group A (with ≥ three factors), Group B (with two factors), and Group C (with ≤ one factor)

(A, B) Show the subgroup analysis of the multifactorial model in treatment lines ≥3 group (n=86). (C, D) Represent the survival outcomes in treatment lines 1-2 group (n=249) based on the multifactorial model. *P*-Values were determined using the log-rank test.

Supplementary Fig. 10 The composition of Group A, Group B, and Group C in the clinically beneficial disease (CD) group and the progressive disease (PD) group

Supplementary Fig. 11 (A) The composition of overall response rate (ORR) and (B) disease control rate (DCR) in Group A, Group B, and Group C, exclusively

Supplementary Fig. 12 Kaplan-Meier curves illustrate overall survival (OS) and progression-free survival (PFS) about the number of favorable baseline factors, which include squamous cell carcinoma, dNLR ≤ 3.18, AMC ≤ 0.48, and IL6 ≤ 12.35. Patients are divided into three groups: Group A (with ≥ three factors), Group B (with two factors), and Group C (with ≤ one factor)

(A, B) Based on the multifactorial model, represent the survival outcomes for the clinically beneficial disease group (n=247). (C, D) Based on the multifactorial model, represent the survival outcomes for the progression disease group (n=88). *P*-Values were determined using the log-rank test.
