# Supplementary material for: Peripheral Blood Biomarkers Predict Outcomes in Advanced Cancers Treated With Anti‐PD‐1 Therapy
Source: Immun Inflamm Dis. 2026 May 6;14(5):e70402. doi: 10.1002/iid3.70402 (PMC13149763; doi:10.1002/iid3.70402)
Supplement: Supplementary file 15 — Supporting File 15 [file IID3-14-e70402-s004.docx]

Supplementary Table 1 The association between Treatment method and clinical categorical variables.

| Characteristics | level | Anti-PD-1 group | Combination Anti-PD-1 group | p | SMD |
| --- | --- | --- | --- | --- | --- |
| N |  | 72 | 263 |  |  |
| Age (%) | ≤73 | 62 (86.1) | 220 (83.7) | 0.745 | 0.069 |
|  | >73 | 10 (13.9) | 43 (16.3) |  |  |
| Gender (%) | woman | 29 (40.3) | 93 (35.4) | 0.529 | 0.102 |
|  | man | 43 (59.7) | 170 (64.6) |  |  |
| ECOG performance status (%) | 1 | 60 (83.3) | 196 (74.5) | 0.16 | 0.217 |
|  | 2 | 12 (16.7) | 67 (25.5) |  |  |
| Primary cancer sites (%) | Biliary duct | 2 (2.8) | 12 (4.6) | 0.003 | 0.675 |
|  | Breast | 0 (0.0) | 4 (1.5) |  |  |
|  | Cervix | 9 (12.5) | 4 (1.5) |  |  |
|  | colorectal | 6 (8.3) | 24 (9.1) |  |  |
|  | Esophagus | 9 (12.5) | 19 (7.2) |  |  |
|  | gastric | 4 (5.6) | 37 (14.1) |  |  |
|  | Head and neck | 7 (9.7) | 28 (10.6) |  |  |
|  | Liver | 9 (12.5) | 31 (11.8) |  |  |
|  | Lung | 14 (19.4) | 67 (25.5) |  |  |
|  | Others | 4 (5.6) | 15 (5.7) |  |  |
|  | Ovary | 0 (0.0) | 3 (1.1) |  |  |
|  | Pancreas | 6 (8.3) | 8 (3.0) |  |  |
|  | Urinary | 2 (2.8) | 11 (4.2) |  |  |
| Histologic subtype (%) | Adenocarcinoma | 33 (45.8) | 155 (58.9) | 0.053 | 0.316 |
|  | Others | 9 (12.5) | 37 (14.1) |  |  |
|  | Squamous carcinoma | 30 (41.7) | 71 (27.0) |  |  |
| Treatment Lines (%) | 1-2 | 52 (72.2) | 197 (74.9) | 0.757 | 0.061 |
|  | ≥3 | 20 (27.8) | 66 (25.1) |  |  |
| Number of metastatic sites (%) | 1-2 | 59 (81.9) | 218 (82.9) | 0.99 | 0.025 |
|  | ≥3 | 13 (18.1) | 45 (17.1) |  |  |
| Bone (%) | Non-metastatic | 51 (70.8) | 196 (74.5) | 0.632 | 0.083 |
|  | Metastasis | 21 (29.2) | 67 (25.5) |  |  |
| Lung (%) | Non-metastatic | 53 (73.6) | 193 (73.4) | 1 | 0.005 |
|  | Metastasis | 19 (26.4) | 70 (26.6) |  |  |
| Liver (%) | Non-metastatic | 56 (77.8) | 182 (69.2) | 0.202 | 0.195 |
|  | Metastasis | 16 (22.2) | 81 (30.8) |  |  |
| Lymph node (%) | Non-metastatic | 30 (41.7) | 139 (52.9) | 0.121 | 0.225 |
|  | Metastasis | 42 (58.3) | 124 (47.1) |  |  |
| Anti-PD-1 cycles (median [IQR]) |  | 8.50 [4.00, 15.25] | 6.00 [4.00, 11.50] | 0.045 | 0.339 |
| Response (%) | CR | 3 (4.2) | 8 (3.0) | 0.409 | 0.22 |
|  | PD | 19 (26.4) | 69 (26.2) |  |  |
|  | PR | 16 (22.2) | 39 (14.8) |  |  |
|  | SD | 34 (47.2) | 147 (55.9) |  |  |

ECOG, Eastern Cooperative Oncology Group; IQR, Interquartile Range; CR, Complete Response; PD, Progressive Disease; PR, Partial Response; SD, Stable Disease; PD, Progressive Disease.

Supplementary Table 2 Univariable and Multivariable Analysis of clinical categorical variables.

| Characteristics | level | Univariable | Multivariable |
| --- | --- | --- | --- |
| Age | >73 y | 1.82 (0.90-3.69, p=.097) |  |
| Treatment Lines | ≥3 (vs. 1-2) | 0.51 (0.25-1.05, p=.066) |  |
| ECOG PS | ≥2 (vs. 0 or 1) | 0.88 (0.45-1.70, p=.703) |  |
| Histologic subtype | Non-squamous carcinoma (vs. Squamous carcinoma) | 2.05 (1.04-4.03, p=.037) | 2.09 (1.03-4.26, p=.041) |
| Number of metastatic sites | Three or more (vs. one or two) | 0.90 (0.43-1.90, p=.787) |  |
| Bone | Metastasis (vs. non-metastatic) | 0.93 (0.50-1.73, p=.811) |  |
| Lung | Metastasis (vs. non-metastatic) | 0.90 (0.48-1.70, p=.755) |  |
| Liver | Metastasis (vs. non-metastatic) | 0.84 (0.45-1.56, p=.576) |  |
| Lymph node | Metastasis (vs. non-metastatic) | 0.88 (0.51-1.53, p=.650) |  |
| Treatment method | Combination anti-PD-1 group (vs. Anti-PD-1 group) | 1.10 (0.79-1.52, p=.566) |  |
| NLR | >4.84 | 1.53 (0.81-2.89, p=.189) |  |
| dNLR | >3.18 | 1.88 (0.97-3.65, p=.061) |  |
| PLR | >290 | 2.52 (1.18-5.40, p=.017) | 1.83 (0.77-4.35, p=.169) |
| ANC | >5.34 | 1.28 (0.65-2.52, p=.480) |  |
| ALC | >1.46 | 0.59 (0.31-1.12, p=.105) |  |
| AMC | >0.48 | 0.77 (0.44-1.34, p=.356) |  |
| IL6 | >12.35 | 2.17 (1.20-3.90, p=.010) | 1.44 (0.73-2.81, p=.291) |
| IL10 | >4.96 | 1.32 (0.57-3.06, p=.518) |  |
| PNI | >44.5 | 0.38 (0.21-0.67, p<.001) | 0.53 (0.21-1.34, p=.178) |
| Serum Albumin | >40.1 | 0.46 (0.26-0.81, p=.007) | 0.99 (0.41-2.39, p=.976) |
| LDH | >250 | 1.74 (0.96-3.17, p=.068) |  |

ECOG PS, Eastern Cooperative Oncology Group Performance Status; NLR, Neutrophil-to-Lymphocyte Ratio; dNLR, Derived Neutrophil-to-Lymphocyte Ratio; PLR, Platelet-to-Lymphocyte Ratio; ANC, Absolute Neutrophil Count; ALC, Absolute Lymphocyte Count; AMC, Absolute Monocyte Count; PNI, Prognostic Nutritional Index; LDH, Lactate Dehydrogenase.

Supplementary Table 3 Multivariable Cox Proportional Hazard Models for PFS and OS in Groups A to C Based on the Number of Favorable Factors.

|  | PFS |  |  | OS |  |  |
| --- | --- | --- | --- | --- | --- | --- |
| Group | HR | 95%CI | P value | HR | 95%CI | P value |
| A | 0.42 | 0.31-0.59 | <0.001 | 0.35 | 0.26-0.48 | <0.001 |
| B | 0.72 | 0.51-1.03 | 0.07 | 0.62 | 0.45-0.86 | <0.001 |
| C | Reference |  |  | Reference |  |  |

Covariables included age (>73 versus ≤73 years), gender (women versus men), Eastern Cooperative Oncology Group performance status (0 or 1 versus 2), histologic subtype (non-squamous carcinoma versus squamous carcinoma), treatment Lines (≥3 versus 1-2), number of metastatic sites (≥3 versus 1-2), metastasis status of bone, lung, liver, and lymph_node (metastasis versus non-metastasis) and mutational peripheral blood parameters (NLR, dNLR, PLR, IL6, IL10, ANC, ALC, AMC, serum albumin, LDH, PNI) were included in multivariable analysis of PFS and OS. PFS, progression-free survival; OS, overall survival; HR, hazard ratio; CI, confidence interval.
